# Supplementary material for: Attitude and predictors of exclusive breastfeeding practice among mothers attending under-five welfare clinics in a rural community in Southwestern Nigeria
Source: PLoS One. 2024 Mar 28;19(3):e0299843. doi: 10.1371/journal.pone.0299843 (PMC10977687; doi:10.1371/journal.pone.0299843)
Supplement: Attachment — Submitted filename: PONE-D-23-40179 (4).pdf_22_12.pdf [file pone.0299843.s002.pdf]

PLOS ONE

Attitude and Predictors of Exclusive Breastfeeding Practice among Mothers Attending Under-Five Welfare Clinics in a Rural Community in Southwestern Nigeria

--Manuscript Draft--

**Manuscript Number:**	PONE-D-23-40179
**Article Type:**	Research Article
**Full Title:**	Attitude and Predictors of Exclusive Breastfeeding Practice among Mothers Attending Under-Five Welfare Clinics in a Rural Community in Southwestern Nigeria
**Short Title:**	Exclusive Breastfeeding Practice among Mothers in Rural Southwestern Nigeria
**Corresponding Author:**	Tope Michael Ipinnimo, MBBS, MPH, FWACP, FMCPH<br>Federal Teaching Hospital, Ido-Ekiti<br>Ido-Ekiti, Ekiti NIGERIA
**Keywords:**	Attitude; exclusive breastfeeding; practice; Nigeria; rural
**Abstract:**	Background: Many previous research on exclusive breastfeeding has focused on urban and semi-urban communities, while there is still paucity of data from the rural areas. We assessed the attitude and practice of exclusive breastfeeding and its predictors among mothers attending the under-five welfare clinics in a rural community.<br>Methods: A cross-sectional study was conducted among consecutively recruited 217 mothers attending the three health facilities under-five welfare clinics in Ido-Ekiti, Southwest, Nigeria. Information was collected with a semi-structured interviewer-administered questionnaire which was adapted from previously published research works. Descriptive and inferential statistics were carried out with the aid of IBM SPSS Statistics for Windows, Version 26.0.<br>Results: More than half of the mothers, 117(53.9%) were ≥30years and 191(88.0%) were married. Almost all, 216 (99.5%) attended an ante-natal clinic however, only 174(80.2%) delivered in the health facility. The mean ± SD exclusive breastfeeding attitudinal score for the respondents was 29.94 ± 2.14 (maximum obtainable scores was 36) and the proportion of mothers that practiced exclusive breastfeeding was 40.6%. Married mothers were more likely to practice exclusive breastfeeding than their unmarried counterparts (AOR:6.324, 95%CI:1.809–22.114). The common reasons for not practicing exclusive breastfeeding were work schedule 57(26.3%), cultural beliefs and the need to introduce herbal medicine 32(14.7%), and insufficient breast milk 30(13.8%).<br>Conclusion: This study revealed a good disposition with a suboptimal practice towards exclusive breastfeeding. Also, being married was a positive predictor of exclusive breastfeeding. Therefore, we recommend policies that will improve exclusive breastfeeding among mothers in rural areas, especially those targeting the unmarried in order to achieve the World Health Organization target.
**Order of Authors:**	Tope Michael Ipinnimo, MBBS, MPH, FWACP, FMCPH
	Olanrewaju Kassim Olasehinde, MBBS, MWACP
	Taofeek Adedayo Sanni, MBBS, MPH, PhD, FWACP, FMCPH
	Ayodeji Andrew Omotoso, MBBS
	Rita Omobosola Alabi, MBBS
	Paul Oladapo Ajayi, MBBS, MPH, FWACP
	Kayode Rasaq Adewoye, MBBS, MPH, MBA, FWACP
	John Olujide Ojo, MBBS, MPH, FWACP
	Olayinka Oloruntoba, MBBS, MWACS
	Blessing Omobolanle Osho
	Adewumi Rufus Fajugbagbe
	Precious Aderinsola Adeyeye

Powered by Editorial Manager® and ProduXion Manager® from Aries Systems Corporation

	Ayotomiwa Fiyinfoluwa Ajayi
**Additional Information:**	
**Question**	**Response**
**Financial Disclosure**<br><br>Enter a financial disclosure statement that describes the sources of funding for the work included in this submission. Review the submission guidelines for detailed requirements. View published research articles from *PLOS ONE* for specific examples.<br><br>This statement is required for submission and **will appear in the published article** if the submission is accepted. Please make sure it is accurate.<br><br>**Funded studies**<br>Enter a statement with the following details:<br>• Initials of the authors who received each award<br>• Grant numbers awarded to each author<br>• The full name of each funder<br>• URL of each funder website<br>• Did the sponsors or funders play any role in the study design, data collection and analysis, decision to publish, or preparation of the manuscript?<br><br>Did you receive funding for this work?	The author(s) received no specific funding for this work.
**Competing Interests**<br><br>Use the instructions below to enter a competing interest statement for this submission. On behalf of all authors, disclose any competing interests that could be perceived to bias this work—acknowledging all financial support and any other relevant financial or non-financial competing interests.<br><br>This statement is required for submission and **will appear in the published article** if the submission is accepted. Please make sure it is accurate and that any funding	The authors have declared that no competing interests exist

Powered by Editorial Manager® and ProduXion Manager® from Aries Systems Corporation

sources listed in your Funding Information later in the submission form are also declared in your Financial Disclosure statement.<br><br>View published research articles from *PLOS ONE* for specific examples.<br><br>**NO authors have competing interests**<br><br>Enter: *The authors have declared that no competing interests exist.*<br><br>**Authors with competing interests**<br><br>Enter competing interest details beginning with this statement:<br><br>*I have read the journal's policy and the authors of this manuscript have the following competing interests: [insert competing interests here]*<br><br>* typeset	
**Ethics Statement**<br><br>Enter an ethics statement for this submission. This statement is required if the study involved:<br><br>• Human participants<br>• Human specimens or tissue<br>• Vertebrate animals or cephalopods<br>• Vertebrate embryos or tissues<br>• Field research<br><br>Write "N/A" if the submission does not require an ethics statement.<br><br>General guidance is provided below. Consult the submission guidelines for detailed instructions. **Make sure that all information entered here is included in the Methods section of the manuscript.**	Ethical approval (ERC/2021/10/18/937A) for this study was obtained from the Human Research and Ethics Review Committee of the Federal Teaching Hospital, Ido-Ekiti, Nigeria. Permission was obtained from the officer-in-charge of the BHC and CHC as well as the consultant-in-charge of the FTH clinics. Additionally, written informed consent to participate was obtained from the mothers prior to the interviews.

**Format for specific study types**<br><br>**Human Subject Research (involving human participants and/or tissue)**<br>• Give the name of the institutional review board or ethics committee that approved the study<br>• Include the approval number and/or a statement indicating approval of this research<br>• Indicate the form of consent obtained (written/oral) or the reason that consent was not obtained (e.g. the data were analyzed anonymously)<br><br>**Animal Research (involving vertebrate animals, embryos or tissues)**<br>• Provide the name of the Institutional Animal Care and Use Committee (IACUC) or other relevant ethics board that reviewed the study protocol, and indicate whether they approved this research or granted a formal waiver of ethical approval<br>• Include an approval number if one was obtained<br>• If the study involved *non-human primates*, add *additional details* about animal welfare and steps taken to ameliorate suffering<br>• If anesthesia, euthanasia, or any kind of animal sacrifice is part of the study, include briefly which substances and/or methods were applied<br><br>**Field Research**<br><br>Include the following details if this study involves the collection of plant, animal, or other materials from a natural setting:<br>• Field permit number<br>• Name of the institution or relevant body that granted permission	
**Data Availability**<br><br>Authors are required to make all data underlying the findings described fully available, without restriction, and from the time of publication. PLOS allows rare exceptions to address legal and ethical concerns. See the PLOS Data Policy and FAQ for detailed information.	Yes - all data are fully available without restriction

Powered by Editorial Manager® and ProduXion Manager® from Aries Systems Corporation

A Data Availability Statement describing where the data can be found is required at submission. Your answers to this question constitute the Data Availability Statement and **will be published in the article**, if accepted.<br><br>**Important:** Stating 'data available on request from the author' is not sufficient. If your data are only available upon request, select 'No' for the first question and explain your exceptional situation in the text box.<br><br>Do the authors confirm that all data underlying the findings described in their manuscript are fully available without restriction?	
**Describe where the data may be found in full sentences. If you are copying our sample text, replace any instances of XXX with the appropriate details.**<br><br>• If the data are **held or will be held in a public repository**, include URLs, accession numbers or DOIs. If this information will only be available after acceptance, indicate this by ticking the box below. For example: *All XXX files are available from the XXX database (accession number(s) XXX, XXX.)*.<br>• If the data are all contained **within the manuscript and/or Supporting Information files**, enter the following: *All relevant data are within the manuscript and its Supporting Information files.*<br>• If neither of these applies but you are able to provide **details of access elsewhere**, with or without limitations, please do so. For example:<br><br>*Data cannot be shared publicly because of [XXX]. Data are available from the XXX Institutional Data Access / Ethics Committee (contact via XXX) for researchers who meet the criteria for access to confidential data.*<br><br>*The data underlying the results presented in the study are available from (include the name of the third party*	All relevant data are within the manuscript and its Supporting Information files

and contact information or URL).<br>• This text is appropriate if the data are owned by a third party and authors do not have permission to share the data.<br><br>* typeset	
Additional data availability information:	

Manuscript

Click here to access/download;Manuscript;MANUSCRIPT - Breatfeeding in Ido-Ekiti.docx

Attitude and Predictors of Exclusive Breastfeeding Practice among Mothers Attending Under-Five Welfare Clinics in a Rural Community in Southwestern Nigeria

Abstract

Background: Many previous research on exclusive breastfeeding has focused on urban and semi-urban communities, while there is still paucity of data from the rural areas. We assessed the attitude and practice of exclusive breastfeeding and its predictors among mothers attending the under-five welfare clinics in a rural community.

Methods: A cross-sectional study was conducted among consecutively recruited 217 mothers attending the three health facilities under-five welfare clinics in Ido-Ekiti, Southwest, Nigeria. Information was collected with a semi-structured interviewer-administered questionnaire which was adapted from previously published research works. Descriptive and inferential statistics were carried out with the aid of IBM SPSS Statistics for Windows, Version 26.0.

Results: More than half of the mothers, 117(53.9%) were ≥30years and 191(88.0%) were married. Almost all, 216 (99.5%) attended an ante-natal clinic however, only 174(80.2%) delivered in the health facility. The mean ± SD exclusive breastfeeding attitudinal score for the respondents was 29.94 ± 2.14 (maximum obtainable scores was 36) and the proportion of mothers that practiced exclusive breastfeeding was 40.6%. Married mothers were more likely to practice exclusive breastfeeding than their unmarried counterparts (AOR:6.324, 95%CI:1.809–22.114). The common reasons for not practicing exclusive breastfeeding were work schedule 57(26.3%), cultural beliefs and the need to introduce herbal medicine 32(14.7%), and insufficient breast milk 30(13.8%).

Conclusion: This study revealed a good disposition with a suboptimal practice towards exclusive breastfeeding. Also, being married was a positive predictor of exclusive breastfeeding. Therefore, we recommend policies that will improve exclusive breastfeeding among mothers in rural areas, especially those targeting the unmarried in order to achieve the World Health Organization target.

Keywords: Attitude, exclusive breastfeeding, practice, Nigeria, rural

Key messages

- The global average of exclusive breastfeeding practice between the years 2015-2021 was 48% while the West Africa region had a prevalence of 35.0% which is 15% less than the World Health Organization recommended target of at least 50.0% in all communities by year 2025.
- This prevalence is even lower among the rural population where the least research that could affect policy and practice on exclusive breastfeeding has been carried out.

- This study which was done in a rural community found that the attitude of the mothers was good, however, the practice of exclusive breastfeeding was still suboptimal. In addition, being married was a positive predictor of exclusive breastfeeding among this population.
- Policies that will improve exclusive breastfeeding among mothers in rural areas were recommended. Furthermore, in-depth research into the factors implicated in the low uptake of exclusive breastfeeding among unmarried women is equally advocated.

Introduction

Breastmilk is described as the ideal food for infants because of its contents and safety. [1] It essentially contains water, nutrients, and antibodies in adequate proportions to promote child growth and development. [1,2] Exclusive breastfeeding (EBF) involves feeding only breastmilk to a child for the first six months of life with the exception of medically prescribed drugs or supplements. [1,3] Optimal nutrition in the first two years of life, including EBF and introduction of nutritionally adequate and safe complementary foods by six months of age, is highly recommended because it has been found to significantly lower morbidity and mortality in the under-five age group. It has also been found to reduce the risk of diarrhoea and chronic disease in children and to encourage better development overall. [2] Breastfeeding not only proves beneficial to child health but also contributes to women's health as well as providing social and economic benefits. [4]

The global average of EBF practice between the years 2015-2021 was 48%. [5] A study done in Italy showed that more than half of the women had heard about EBF with a majority of them believing that EBF is important to both mother and child. [6] More than 90% had breastfed their children but only 33.3% had practiced EBF. [6] Another study in Bangladesh, which focused on mothers in rural areas, showed poor knowledge (34.5%) and practice (27.9%) of EBF. [7] A systematic review of studies carried out in East Africa showed that 42% of mothers preferred to feed their infants with breastmilk alone for the first six months of life while 55.9% of them had practiced EBF for at least six months. [8] The West Africa region had a prevalence of 35.0% for exclusive breastfeeding between the years 2015-2021, one of the lowest in the world for the same period. [5]

In Nigeria, only 28.7% of babies were exclusively breastfed in the year 2018. [9] This was even lower in the rural population with a report of 24.3%. [9] A study in a semi-urban area of Sokoto state, Northwest Nigeria, indicated that only 31.0% of women practiced EBF. [10] Another study carried out in one of the urban centers in Abuja, the Federal Capital Territory of Nigeria showed that 54.4% had practiced EBF despite a remarkable positive attitude that was determined by the study (70% agreed that EBF was adequate for their children). [11] A similar study in another urban center in Benin, Edo state, South-south Nigeria, showed an "ever-breastfed rate" of 100.0% but only 40.7% practiced EBF. [12] A survey carried out in two tertiary hospitals in

Ogun state, Southwest Nigeria, revealed that more than half (58.8%) of the women practiced EBF. [13]

Optimal breastfeeding practices are important in the prevention and management of malnutrition in children. [2] Stunting, which is a form of malnutrition and an indication of poor nutrition beginning in-utero into early childhood, affected an estimated 144 million (21.3%) under-five children globally in 2019 with the highest burdens in Africa and Asia continents. [14] Over one-third (37.0%) of Nigerian children aged 6 - 59 months are stunted and the prevalence of stunting is almost twice as high among children in rural areas (45.0%) as among those in urban areas (27.0%). [9]

Many previous studies on EBF have centered on urban and semi-urban communities while there is still a paucity of data from the rural areas. [10-13] To the best of the researchers' knowledge, there has not been a study of this nature assessing the attitude and practice of mother on EBF in the environment. This study aims to determine the attitude, practice and identify the predictors of practice of EBF among mothers attending the under-five welfare clinics in a rural community in Southwest Nigeria. The findings of this study would contribute to the literature and can help facilitate further research on the subject. Also, it would aid in defining the gaps in women's attitude to EBF and its practice, especially in the rural areas. This could then serve as a basis for health planning, policy formulation and implementation to improve EBF practice in rural Nigeria in order to meet the World Health Organization (WHO) recommended target of at least 50.0% EBF rate in all communities by 2025. [15]

Methods

Study area and design

This was a cross-sectional study carried out between 1st and 30th November 2021 in all the under-five welfare clinics in Ido-Ekiti, Southwest, Nigeria. Ido-Ekiti is a town with an estimated population of 37,000. [16] There are three health facilities with under-five welfare clinic in the community which are the Basic Health Centre (BHC), Comprehensive Health Centre (CHC) and the Federal Teaching Hospital (FTH), Ido-Ekiti. The BHC and CHC are primary health facility each with a staff strength of less than thirty healthcare workers mainly registered nurses/midwives and community health extension workers. The BHC and CHC under-five welfare clinics provides services such as immunization and growth monitoring on clinic days which is just once in a week and each of them sees an average of 10 to 20 children per day in their clinics. The FTH is a tertiary health facility that serves as a referral centre for other health facilities within the environs. The under-five welfare clinic in FTH is located at the Department of Community Medicine of the hospital and it offers services every day with the exception of weekends (Saturday to Sunday) to an average of 20 children per day. The staff in the clinic consists of community/public health physicians, public health nurses and community health extension workers.

Participants, sample size determination and sampling technique

The study population consisted of mothers attending the under-five welfare clinics in the community. The study included all healthy mothers with healthy baby(ies) attending the clinics. Mothers with adopted or fostered babies were excluded from the study. The minimum sample size for the study was determined using Leslie Fisher's formula. [17] A 95% confidence interval, and 5% degree of accuracy was assumed. A sample size of 217 was gotten after using the prevalence of EBF from a previous study. [18] All the three health facilities were used for the study. The number of mothers selected from the health facilities was determined using proportionate allocation based on the under-five welfare clinic attendance. Eligible mothers attending the clinic were recruited consecutively into the study until the sample size was achieved.

Data Collection Methods

The study instrument used to collect information from the women is a 26-item semi-structured interviewer-administered questionnaire which was adapted from previously published research works. [19, 20] The questionnaire obtained data on socio-demographic and other (such as antenatal clinic attendance, place of delivery) variables, attitude towards EBF, practice of EBF and reasons for not breastfeeding exclusively. The questionnaire was pre-tested on a sample (twenty) of mothers in a CHC in another community which were neither analyzed nor included in the study. Necessary adjustments and corrections were made to the questionnaire after the pre-test. The questionnaire administration was done by the researchers in a scheduled area of the health facilities through face-to-face interviews with the mothers immediately after their clinics.

Data management and statistical analysis

The data collected were entered, cleaned and analysed using computer software IBM SPSS (IBM SPSS Statistics for Windows, Version 26.0. Armonk, NY: IBM Corp). Data were summarized in tables using frequencies and percentages. There were twelve questions that assessed the attitude of mothers on EBF. Mothers were asked to rate their responses on a 3-point Likert scale measuring the intensity of mother's attitudes. [19] The attitudinal scores of the participants were calculated and presented using mean and standard deviation. The mean attitudinal scores across each socio-demographic and other variables category were compared using the independent Student-T-test. The association between mother's characteristics and the practice of EBF was assessed using Chi-square test. Binary logistic regression analysis was used to determine the predictors of EBF. P-values <0.05 was considered significant for the inferential statistics.

Ethical Considerations

Ethical approval (ERC/2021/10/18/937A) for this study was obtained from the Human Research and Ethics Review Committee of the Federal Teaching Hospital, Ido-Ekiti, Nigeria. Permission was obtained from the officer-in-charge of the BHC and CHC as well as the consultant-in-charge of the FTH clinics. Additionally, written informed consent to participate was obtained from the mothers prior to the interviews.

Results

A total of 217 mothers completed the survey questionnaire. Table 1 shows that more than half of the mothers, 117 (53.9%) were ≥ 30 years and more than two-thirds, 148 (68.2%) held a post-secondary school degree. More than three-quarters, 191(88.0%) and 179 (82.5%) were married and of Yoruba ethnicity respectively. Ninety-four (43.3%) of them were civil servants. The majority of the mother, 216 (99.5%) attended ante-natal clinic, out of which 214 (98.6%) were informed on EBF. Most, 174 (80.2%) delivered within a health facility and 182 (83.9%) deliveries were via vaginal delivery. More, 91 (41.9%) of the mothers are currently nursing their first child.

Table 2 show that the mean (standard deviation) EBF attitude score for all respondents was 29.94 (2.14) out of 36 maximum possible scores. EBF attitude scores significantly differed across age ($T = -2.844$, $p = 0.005$), level of education ($T = -2.273$, $p = 0.025$), ante-natal clinic attendance during the pregnancy of the baby ($T = 4.895$, $p < 0.001$), been informed on EBF during ante-natal clinic ($T = 14.378$, $p < 0.001$) and the birth order of the child ($F = 9.533$, $p = 0.001$). Attitudinal score did not significantly differ across other variables.

Eighty-eight (40.6%) of the mothers practiced EBF and there was a statistically significant association between marital status and practice of EBF ($X = 8.993$, $p = 0.003$). Higher proportion of married mothers practiced EBF than unmarried mothers (married: 44.5%; unmarried: 11.5%). There was no statistically significant association between other patient variables and practice of EBF. Binary logistic regression in table 4 revealed that mothers who are married were about 6 time more likely to practice EBF than those who are unmarried (AOR:6.324, 95%CI: 1.809 – 22.114).

According to table 5, the reasons for the mothers not practicing EBF were work schedule 57 (26.3%), cultural beliefs and the need to introduce herbal medicine 32 (14.7%), insufficient breast milk 30 (13.8%), baby gets hungry and thirsty 28 (12.9%), HIV and unplanned pregnancy 19 (8.8%), early and single motherhood 14 (6.5%) and others reasons 4 (1.8%).

Discussion

This study assessed the attitude towards EBF, its practice, and predictors among mothers attending under-five welfare clinics in a rural community. The mean attitude score towards EBF for all mothers in this study was 29.9 out of 36 maximum possible scores. This is 83.2% of the maximum obtainable score which depicts a good disposition towards EBF among the mothers. This finding is similar to what was found in studies done among lactating mothers in a rural Ghana community and in Ethiopia. [19, 20] This level of attitude is good for the mothers, although there is room for improvement.

This study revealed that EBF attitude is positively associated with age, as mothers who were 30 years and over had a better attitude than those aged less than 30 years. This finding is consistent with findings from studies in Ondo state, Southwest, Nigeria [21] and in the Island of Abu Dhabi, United Arab Emirates. [22] Also, mothers who had attended antenatal clinic during the pregnancy of the baby had a better attitude than mothers who did not attend the clinic. The antenatal clinic provides nutritional counseling and education including the health benefits of EBF and other infant feeding practices as part of the health promotion measures during visits.

Unsurprisingly, this exposure not only improves maternal attitude but subsequently brings about the likelihood of better practice as reported in a study done in Rawalpindi. [23]

This study found that mothers with a higher level of education had better dispositions toward EBF. Maternal level of education is a significant determinant of infant feeding practices, and this is supported by a study done in Ondo state, Southwest, Nigeria which found that attitude towards EBF is higher among lactating mothers with higher levels of education. [21] Mothers with higher levels of education can understand and quickly realize the benefits of EBF to their infants and are more motivated to practice it. Programs that promote EBF should be adapted to appeal to mothers who have lower levels of education. The maternal attitude of EBF was better among mothers with 2nd birth than those with their first birth. This is similar to the findings in a study done in Ibadan, Southwest, Nigeria. [24] This finding is not surprising as multiparous mothers have previously developed a prior experience and skillset which allows for better EBF attitude in subsequent deliveries.

Less than half (40.6%) of mothers in this study practiced EBF. This is similar to a study done in Edo state, South-south, Nigeria with a prevalence of 40.7%. [12] However, it is higher than the findings in previous studies done in Italy (33.3%), Bangladesh (34.5%) and Sokoto state, Northwest, Nigeria (31.0%) [6,7,10] and lower than the prevalence recorded from studies conducted in urban centers of Abuja (54.4%) and in Ogun State, Southwest, Nigeria (58.8%). [11, 13] Interestingly, the prevalence of practice of EBF in this study aligns with the WHO targets of at least 50.0% rate in all communities whether rural or urban by 2025. [15] Though, close to the WHO target, several barriers still hinder the practice of EBF. Work schedule, cultural beliefs and the need to introduce herbal medicine, insufficient breast milk, baby gets hungry and thirsty, HIV and unplanned pregnancy, early and single motherhood were mentioned by the mothers as barriers to EBF. Some of these barriers have equally been reported in previous studies. [25, 26]

It was observed from this study that mothers who are married were about 6 times more likely to practice EBF than those who are unmarried. Expectedly, this result is substantiated by another study in Ethiopia. [27] This may be due to the responsibility-sharing and support provided by the husband that could aid the practice of EBF. However, this finding contrast that found in a study done among first-time mothers in Ethiopia which revealed that participants who were not married were almost 3-fold more likely to practice EBF compared with married counterpart. [28] This difference could be due to birth-related traditional practices of the Ethiopian people where first-time mothers at 8 months gestational age visit their parent's home in preparation for births, and afterward stay for 40 days after birth with their infant and never left alone. Against these background, one can deduce that a good support system from husbands of married women or from parents of unmarried women post-delivery makes the practice of EBF more feasible.

This study shares the limitations of the cross-sectional study design. In addition, this research is prone to social desirability and recall bias since questions were asked about the past experiences of the mothers on breastfeeding. Lastly, this is a health facility-based study that was carried out in the under-five welfare clinic and might have left out mothers who do not seek healthcare or

that patronize other institutions such as patent medicine store, drug hawkers and herbalist for their healthcare.

In conclusion, the study revealed a good disposition towards EBF among mothers with less than half (40.6%) breastfeeding their babies exclusively. The common reasons given for not breastfeeding exclusively included work schedule, cultural beliefs and the need to introduce herbal medicine, insufficient breast milk and baby gets hungry and thirsty. Also, it was found that the married mothers were more likely to practice EBF than unmarried mothers. We therefore recommend policies that will improve the EBF of mothers in rural areas toward achieving the WHO target. It is also important to carry out further quantitative and qualitative studies to identify factors associated with low uptake of EBF among unmarried women and develop intervention to improve their practice.

Competing Interest:

Authors declare they have no competing interest in conducting this study

References

1. World Health Organization. Breastfeeding: Questions and Answers. 2015. Available from: https://www.who.int/news-room/questions-and-answers/item/breastfeeding [Last accessed 8 February 2023]

2. World Health Organization. Infant and young child feeding. 2021. Available from: https://www.who.int/news-room/fact-sheets/detail/infant-and-young-child-feeding [Last accessed 8 February 2023]

3. Centre for Disease Control and Prevention. Nutrition: Infant and Toddler nutrition. 2022. https://www.cdc.gov/nutrition/infantandtoddlernutrition/definitions.html [Last accessed 8 February 2023]

4. The Innocenti declaration on the protection, promotion, and support of breastfeeding. 1990. Florence, Italy. World Health Organization/United Nation Children's Fund.

5. United Nation Children's Fund. Global Database for Exclusive Breastfeeding. 2022. Available from: https://data.unicef.org/topic/nutrition/breastfeeding/ [Last accessed 8 February 2023]

6. Cascone D, Tomassoni D, Napolitano F, Di Giuseppe G. Evaluation of Knowledge, Attitudes, and Practices about Exclusive Breastfeeding among Women in Italy. Int J Environ Res Public Health. 2019;16(12):2118. DOI: 10.3390/ijerph16122118.

7. Rana MM, Islam MR, Karim MR, Islam AZ, Haque MA, Shahiduzzaman M, Hossain MG. Knowledge and practices of exclusive breastfeeding among mothers in rural areas of Rajshahi district in Bangladesh: A community clinic-based study. PLoS One. 2020;15(5):e0232027. DOI: 10.1371/journal.pone.0232027.

8. Dukuzumuremyi JPC, Acheampong K, Abesig J, Luo J. Knowledge, attitude, and practice of exclusive breastfeeding among mothers in East Africa: a systematic review. Int Breastfeed J. 2020;15(1):70. DOI: 10.1186/s13006-020-00313-9.

9. National Demographic and Health Survey. 2018. National Population Commission. Federal Republic of Nigeria.

10. Oche MO, Umar AS, Ahmed H. Knowledge and practice of exclusive breastfeeding in Kware, Nigeria. African Health Sciences 2011; 11(3): 518 – 523

11. Okoroiwu GIA, Ubosi NI, Aliyu SM, Eya CP. Knowledge, Attitude and Practice of Exclusive Breastfeeding amongst Mothers of Infants in Gwagwalada Area Council, FCT, Abuja, Nigeria. J. Appl. Sci. Environ. Manage. 2021;25(1):127-132. https://dx.doi.org/10.4314/jasem.v25i1.18

12. Akpan UJ, Ibadin MO, Abiodun PO. Breastfeeding practices in early infancy in Benin City, Nigeria. Niger J Paed. 2015;42(2): 126 –131. http://dx.doi.org/10.4314/njp.v42i2.11

13. Adebayo A, Oluwaseyi O. Breastfeeding Practices among Mothers in Southwest Nigeria. Ethiop J Health Sci. 2020;30(5):697. http://dx.doi.org/ 10.4314/ejhs.v30i5.8

14. United Nations Children's Fund, World Health Organization & World Bank. 2020. Levels and trends in child malnutrition: UNICEF/WHO/The World Bank Group joint child malnutrition estimates: key findings of the 2020 edition. World Health Organization. Available from: https://apps.who.int/iris/handle/10665/331621 [Last accessed 4 March 2023]

15. World Health Organization. Global nutrition targets 2025: Policy brief series. 2014. Available from: https://www.who.int/publications/i/item/WHO-NMH-NHD-14.2 [Last accessed 4 March 2023]

16. City-facts. Ido-Ekiti. 2015. Available from: https://www.city-facts.com/ido-ekiti [Last accessed 4 March 2023]

17. Ipinnimo TM, Abodunrin OL, Durowade KA. Relationship between Cost of Care and Medication Adherence among Hypertensive Patients attending a Tertiary Health Facility in Ekiti State, Nigeria. Journal of community medicine and primary health care. 2022; 34(1):38-57. https://dx.doi.org/10.4314/jcmphc.v34i1.3

18. Adewuyi EO, Adefemi K. Breastfeeding in Nigeria: a systematic review. Int J Community Med Public Health. 2016;3(2):385-396. http://dx.doi.org/10.18203/2394-6040.ijcmph20160421

19. Mogre, V., Dery, M., Gaa, P.K. Knowledge, attitudes and determinants of exclusive breastfeeding practice among Ghanaian rural lactating mothers. Int Breastfeed J. 2016;11:12. https://doi.org/10.1186/s13006-016-0071-z

20. Tadele N, Habta F, Akmel D, Deges E. Knowledge, attitude and practice towards exclusive breastfeeding among lactating mothers in Mizan Aman town, Southwestern Ethiopia: descriptive cross-sectional study. Int Breastfeed J. 2016;11:3. DOI: 10.1186/s13006-016-0062-0.

21. Adegboro JS. Influence of demographic variables on attitude of nursing mothers towards exclusive breastfeeding in Akoko South West Local Government Area of Ondo State, Nigeria. International Journal of Life Science Research Archive. 2022;3(1):031-8. https://doi.org/10.53771/ijlsra.2022.3.1.0065

22. Al Ketbi MI, Al Noman S, Al Ali A, Darwish E, Al Fahim M, Rajah J. Knowledge, attitudes, and practices of breastfeeding among women visiting primary healthcare clinics on the island of Abu Dhabi, United Arab Emirates. Int Breastfeed J. 2018;13:26. DOI: 10.1186/s13006-018-0165-x.

23. Ahmad MO, Sughra U, Kalsoom U, Imran M, Hadi U. Effect of antenatal counseling on exclusive breastfeeding. J Ayub Med Coll Abbottabad. 2012;24(2):116-9.

24. Ayinde AO, Adedokun B, Adeloye AI, Agbonjimi LA, Ayinde TO, Ogunode NJ. Birth order and exclusive breastfeeding practices among mothers with two or more children. Ijcsmr. 2021;1(1):1-10. https://journalofmedical.org/index.php/ijcsmr

25. Otoo GE, Lartey AA, Pérez-Escamilla R. Perceived incentives and barriers to exclusive breastfeeding among periurban Ghanaian women. Journal of Human Lactation. 2009;25(1):34-41. doi: 10.1177/0890334408325072.

26. Apanga PA. A review on facilitators and barriers to exclusive breastfeeding in West Africa. Journal of Biology, Agriculture and Healthcare. 2014;4:9–15.

27. Mamo K, Dengia T, Abubeker A, Girmaye E. Assessment of Exclusive Breastfeeding Practice and Associated Factors among Mothers in West Shoa Zone, Oromia, Ethiopia. Obstet Gynecol Int. 2020;2020:3965873. doi: 10.1155/2020/3965873.

28. Ayalew T. Exclusive breastfeeding practice and associated factors among first-time mothers in Bahir Dar city, North West Ethiopia, removed: A community based cross sectional study. Heliyon. 2020;6(9):e04732. doi:10.1016/j.heliyon.2020.e04732

Tables

Table 1 Socio-demographic and other characteristics of the respondents

Variable	Frequency (N= 217)	Percent (%)
**Age**		
< 30 years	100	46.1
≥ 30 years	117	53.9
**Level of education**		
Secondary	69	31.8
Post-secondary	148	68.2
**Marital status**		
Unmarried	26	12.0
Married	191	88.0
**Religion**		
Christianity	174	80.2
Islam	43	19.8
**Tribe**		
Yoruba	179	82.5
Hausa	16	7.4
Igbo	22	10.1
**Occupation**		
Farmer/trader/artisan	76	35.0
Civil servant	94	43.3
Professional	20	9.2
Student	27	12.4
**ANC attendance during the pregnancy of this baby**		
Yes	216	99.5
No	1	0.5
**If yes, were you informed on exclusive breastfeeding (n=216)**		
Yes	214	98.6
No	2	0.9
**Place of delivery of this baby**		
Outside health facility	43	19.8
Within health facility	174	80.2
**Mode of delivery of this baby**		
Vaginal delivery	182	83.9
Caesarean section	35	16.1
**Birth order of the baby**		
First	91	41.9
Second	75	34.6
3 and above	51	23.5

Table 2 Association between mothers' characteristics and exclusive breastfeeding attitude score

Variable	Attitude score Mean ± SD	Test	p-value
**All respondents**	29.94 ± 2.14	-	-
**Age (in years)**		-2.844^{T}	**0.005**
< 30 years	29.50 ± 1.956		
≥ 30 years	30.30 ± 2.226		
**Level of education**		-2.273^{T}	**0.025**
Secondary	29.43 ± 2.29		
Post-secondary	30.16 ±2.02		
**Marital status**		-0.480^{T}	0.634
Unmarried	29.76 ± 1.839		
Married	29.95 ± 2.180		
**Religion**		1.974^{T}	0.053
Christianity	30.09 ± 2.007		
Islam	29.27 ± 2.529		
**Tribe**		0.317^{F}	0.729
Yoruba	29.97 ± 2.075		
Hausa	30.00 ± 1.591		
Igbo	29.59 ± 2.938		
**Occupation**		0.427^{F}	0.734
Farmer/trader/artisan	29.72 ± 2.194		
C/S	30.09 ± 2.374		
Professional	30.00 ± 2.000		
Student	29.92 ± 0.196		
**ANC attendance during the pregnancy of this baby**		4.895^{T}	**<0.001**
Yes	29.98 ± 2.034		
No	20.00 ± 0.000		
**If yes, were you informed on exclusive breastfeeding**		14.378^{T}	**<0.001**
Yes	30.00 ± 2.034		
No	28.00 ± 0.000		
**Place of delivery**		0.571^{T}	0.570
Outside health facility	30.11 ± 2.372		
Within health facility	29.98 ± 2.083		
**Mode of delivery**		-2.773^{T}	0.008
Vaginal delivery	29.78 ± 2.166		
Caesarean section	30.74 ± 1.820		
**Birth order**		9.533^{F}	**0.001**
First	29.45 ± 2.088		
Second	30.77 ± 1.713		
Third and above	29.56 ± 2.443		

SD: Standard deviation, ^{T}: Student T test, ^{F}: Analysis of variance

Table 3 Association between mothers' characteristics and practice of exclusive breastfeeding

Variable	Exclusive breastfeeding Yes (n=88) (%)	No (n=129) (%)	Test	p-value
**Age group (years)**			0.015^{X}	1.000
<30 years	41 (41.0)	59 (59.0)		
>30 years	47 (40.2)	70 (59.8)		
**Level of education**			0.091^{X}	0.769
Secondary	29 (42.0)	40 (58.0)		
Post-secondary	59 (39.9)	89 (60.1)		
**Marital status**			8.993^{Y}	**0.003**
Unmarried	3 (11.5)	23 (88.5)		
Married	85 (44.5)	106 (55.5)		
**Religion**			0.249^{X}	0.729
Christianity	72 (41.4)	102 (58.6)		
Islam	16 (37.2)	27 (52.8)		
**Tribe**			3.506^{F}	0.173
Yoruba	75 (49.1)	104 (58.9)		
Hausa	3 (18.8)	13 (81.3)		
Igbo	10 (45.5)	12 (54.5)		
**Occupation**			2.257^{X}	0.529
Farmer/trader/artisan	32 (42.9)	44 (57.1)		
C/S	34 (36.2)	60 (63.8)		
Professional	8 (40.0)	12 (60.0)		
Student	14 (51.9)	13 (48.1)		
**ANC attendance during the pregnancy of this baby**			$<0.001^{Y}$	1.000
Yes	88 (40.7)	128 (59.3)		
No	0 (0.0)	1 (100.0)		
**If yes to question 7, were you informed on exclusive breastfeeding**			0.207^{Y}	0.649
Yes	88 (41.1)	126 (58.9)		
No	0 (0.0)	2 (100.0)		
**Place of delivery of this baby**			0.715^{X}	0.488
Outside HF	15 (34.9)	28 (65.1)		
Within HF	73 (42.0)	101 (58.0)		
**Mode of delivery**			0.005^{X}	1.000
Vaginal delivery	74 (40.7)	108 (59.3)		
Caesarean section	14 (40.0)	21 (60.0)		
**Birth order**			2.859^{X}	0.245
First	32 (35.2)	59 (54.8)		
Second	36 (48.0)	39 (52.0)		
Third and above	20 (39.2)	31 (60.8)		
**Mean attitude score**	30.09 ± 1.891	29.82 ± 2.295	0.883^{T}	0.378

^{X}: Pearson Chi-Square, ^{Y}: Continuity correction, ^{F}: Fisher's Exact Test

Table 4 Predictors of practice of excusive breastfeeding

Predictors				95% CI for AOR	
	B	P-value	AOR	Lower	Upper
**Marital Status (**Married)	1.844	**0.004**	6.324	1.809	22.114
**Tribe** (Hausa)	0.357	0.459	1.429	0.556	3.674
**Tribe** (Igbo)	1.429	0.072	4.173	0.879	19.803

B: Intercept, AOR: Adjusted odd ratio, CI: Confidence interval

Table 5 Reasons for not practicing exclusive breastfeeding

Reasons for not practicing exclusive breastfeeding*	Frequency (N=217)	Percent (%)
The baby gets hungry and the child being thirsty	28	12.9
HIV and unintended pregnancies	19	8.8
Breast milk was insufficient	30	13.8
Work schedule	57	26.3
Early and single motherhood	14	6.5
Cultural belief and the need to introduce herbal medicine	32	14.7
Others	4	1.8

*Multiple response
