# Supplementary material for: Attitude and predictors of exclusive breastfeeding practice among mothers attending under-five welfare clinics in a rural community in Southwestern Nigeria
Source: PLoS One. 2024 Mar 28;19(3):e0299843. doi: 10.1371/journal.pone.0299843 (PMC10977687; doi:10.1371/journal.pone.0299843)
Supplement: Attachment — Submitted filename: Response to Reviewers - Breastfeeding in Ido-Ekiti.doc [file pone.0299843.s004.doc]

**REPLY TO THE REVIEWERS’ COMMENTS**

**Manuscript title:** Attitude and Predictors of Exclusive Breastfeeding Practice among Mothers Attending Under-Five Welfare Clinics in a Rural Community in Southwestern Nigeria

**Manuscript ID:** PONE-D-23-40179

Dear Academic Editor and Reviewers,

Thank you for taking the time to read and review this work, we are grateful for your insightful comments and contributions. Please find the response to each point raised in the table below.

| **Reviewer Number** | **Original comments of the reviewer** | **Reply by the author(s)** |
| --- | --- | --- |
| REVIEWER 1 | • You can include key messages in your final conclusion section | This has been moved to the conclusion section |
|  | • The definition of breastmilk should be rewrite again.  • There is a reference repetition.  • This is more to the problem situation and it has to be coherent; revise the background section  • You didn't show the actual gap of the study area in this section | The definition of breastmilk has been rewritten  Reference repetition has been adjusted  The background section has been revised as suggested and the gap in the study area has been highlighted. |
|  | • The multivariate regression is don’t exist to the extent to identify the predictors  • The legend of the table description should clearly show the data  • It is good to display the multivariate data of what you have stated right here | The multivariate logistic regression was conducted following bivariate analysis. Variable with p ≤ 0.2 at the bivariate (Chi square) level were included and used to build the logistic model.  The legend of table has been revised as suggested.  A graphical method has been employed to display the data. |
|  | • The discussion is not enough. You have to revise additional and similar topics in Ethiopia and other countries you have stated In General, at this point I can give these comments. | The discussion section has been revised and additional literature from Ethiopia and Nigeria has been included.  Thank you for your comments and suggestions. |
|  |  |  |
| REVIEWER 2 | The title has been over researched in Nigeria, so what did you add to the existing knowledge or what is your new finding? If you can justify this, what about the knowledge component? it should be included in the title or studied as a factor The introduction section should clearly describe the public health importance of EBF and mother's attitude, it should be based on your objectives, and must show what interventions undertaken and the existing gaps | To the best of our knowledge, this is the first study in Ekiti state that assessed the attitude and practice of exclusive breastfeeding among rural mothers. We also identified predictors of exclusive breastfeeding among this population.  Thank you for the suggestion. The Introductory section has been revised as suggested. |
|  | Regarding to sample size, your sample size is not adequate, and since the sampling technique is non random, it can not be used for generalization. How do you measure attitude and practice? I think you used mean, what was your reason to use the mean? I haven't seen a clear operational definition. In addition to this using a five point Likert scale is better than 3 point Liker Scale. It is better to show the dependent and independent variables clearly. Regarding to ethical issue, how did you address the confidentiality issue? | Thank you for the comment. We used Leslie Fisher’s formula to estimate the minimum sample size and obtained 217. This was proportionately allocated to the facilities involved based on their client strength. However, respondents were recruited consecutively over the study period and this has been detailed in the method section.  An explanation on how “attitude” and “practice” were measured and analyzed has been added under the new subsection named “Data collection tool” within the methods.  Dependent and independent variables are now clearly stated under “Variables” subsection.  Confidentiality and anonymity were maintained by not collecting personal data such as names and phone numbers from the respondents. This statement has been added to the “Ethical Consideration subsection” |
|  | Result: the statistical analysis is not performed appropriately and adequately, and lacks clarity. For example you said "The mean ± SD exclusive breastfeeding attitudinal score for the respondents was 29.94 ± 2.14 (maximum obtainable scores was 36) and the proportion of mothers that practiced exclusive breastfeeding was 40.6%." What are these numbers? showing the confidence interval is significant. The attitude score is not is not clear, you put only the mean score in table 2 there should be a table which shows the attitude components. Marital status is the only variable associated with EBF practice, but what were the other variables in binary logistic regression which entered into multivariable logistic regression? | Twelve questions assessed the attitude of mothers on EBF. Mothers were asked to rate their responses on a 3-point Likert scale (1 to 3) measuring the intensity of mother’s attitudes. A positive response was rated 3, a neutral rated 2, and a negative rated 1. The attitudinal score of each respondent was estimated by summing their rating with a maximum obtainable score of 36. The attitudinal scores of all the participants and across different variable categories were calculated and presented using mean and standard deviation.  Exclusive breastfeeding practice was assessed with a question inquiring if mothers have given only breast milk to the baby for the first 6 months of life without infant formula or any other product unless it was prescribed by a physician. This was a dichotomous variable (“Exclusively breastfed” OR “Not exclusively breastfed”).  The logistic regression model was built with variables with p ≤ 0.2 at the bivariate (Chi-square test) level of analysis and these variables included marital status as well as tribe. This is presented in Table 4 |
|  | Discussion and conclusion should be objective based, and the recommendation should be based on your finding. | Thank you for the comment. This has been revised as suggested. |
|  | Additionally, I am not sure that the referencing style fulfils PLOS ONE's criteria. | We have double-checked this to fulfil the PLOS ONE’s criteria |
|  |  |  |
| REVIEWER 3 | The study topic is scientifically sound and may contribute to body knowledge in maternal and child health. However, there are many gross editorial and methodological issues and problems. I have tracked and changed all the comments and suggestions with an attached document. 1. The study was written in standard academic English. | Thank you for the comments. All the corrections under track changes have been made. In addition, we have revised other editorial and grammatical corrections. |
|  | 2. The methodology needs a massive correction. You could not provide a specific Analysis that fits the nature of your data. a. You must have separate titles for data collection methods, tools, or study measurements. They are district features of the study methodology. b. If you did a pre-test, what was the tool's reliability? c. you need to state explicitly how you measured the exclusive breastfeeding outcome variable. d. What is the need to use chi-square? Do you have a specific justification? You also used binary logistic regression, which measures the association between dependent and independent variables more accurately than chi-square. Table 2 also indicates you used Yate’s continuity correction and Fisher’s exact test. e. Did you conclude the association between dependent and independent variables only based on the result of binary logic regression? This is not scientifically sound.  Table 1 depicts. You used Analysis of variance and T-test. So, you did not select one appropriate analysis method for this study. If you have justification, why do you use a mix of different analyses to justify it? | This methods section has been revised based on your suggestions.  Data collection method is now separate from the Study instrument.  Tool reliability has been included.  An explanation on how “attitude” and “practice” were measured and analyzed has been added under the new subsection named “Data collection tool” within the methods.  The Chi-square test was used to compare the association between the practice of EBF and sociodemographic/maternal variables at the bivariate level of analysis. It was used because they were both categorical variables and Chi-square is used to test for association between two categorical variables. The Yate correction and Fisher’s exact test were used in situations where we have observation in cell(s) < 5 as shown that the Chi-square test may not be the best test in such situations.  The binary logistic regression analysis was used at the multivariate level of analysis to determine the predictors of EBF. The logistic regression model was built with variables with p ≤ 0.2 at the bivariate (Chi-square test) level of analysis.  Student-T-test and ANOVA (F-test) were used to compare the attitudinal scores as these were continuous variables (mean and SD). T test was for comparing 2 groups while ANOVA was for >2 groups. |
|  | 3. You need to add the strengths and limitations of the study and the conclusion and recommendation sections. | Done as suggested |
