# Supplementary material for: Attitude and predictors of exclusive breastfeeding practice among mothers attending under-five welfare clinics in a rural community in Southwestern Nigeria
Source: PLoS One. 2024 Mar 28;19(3):e0299843. doi: 10.1371/journal.pone.0299843 (PMC10977687; doi:10.1371/journal.pone.0299843)
Supplement: Attachment — Submitted filename: PONE-D-23-40179_R1_reviewer Commented second.docx [file pone.0299843.s005.docx]

PLOS ONE

Attitude and Predictors of Exclusive Breastfeeding Practice among Mothers Attending Under-Five Welfare Clinics in a Rural Community in Southwestern Nigeria

--Manuscript Draft--

| Manuscript Number: | PONE-D-23-40179R1 |
| --- | --- |
| Article Type: | Research Article |
| Full Title: | Attitude and Predictors of Exclusive Breastfeeding Practice among Mothers Attending Under-Five Welfare Clinics in a Rural Community in Southwestern Nigeria |
| Short Title: | Exclusive Breastfeeding Practice among Mothers in Rural Southwestern Nigeria |
| Corresponding Author: | Tope Michael Ipinnimo, MBBS, MPH, FWACP, FMCPH Federal Teaching Hospital, Ido-Ekiti  Ido-Ekiti, Ekiti NIGERIA |
| Keywords: | attitude; Exclusive breastfeeding; Practice; Nigeria; rural |
| Abstract: | Background: Much previous research on exclusive breastfeeding has focused on urban and semi-urban communities, while there is still a paucity of data from rural areas. We assessed the attitude and practice of exclusive breastfeeding and its predictors among mothers attending the under-five welfare clinics in a rural community. Methods: A cross-sectional study was conducted among consecutively recruited 217 mothers attending the three health facilities under-five welfare clinics in Ido-Ekiti, Southwest, Nigeria. Information was collected with a semi-structured interviewer- administered questionnaire adapted from previously published research works. Descriptive and inferential statistics were carried out using IBM SPSS Statistics for Windows, Version 26.0.  Results: More than half of the mothers, 117(53.9%) were ≥30 years old, and 191(88.0%) were married. Almost all 216 (99.5%) attended an ante-natal clinic and 216 (99.5%) attended an ante-natal clinic; however, 174(80.2%) delivered in the health facility. The respondent’s mean ± SD exclusive breastfeeding attitudinal score was 29.94 ± 2.14 (maximum obtainable scores was 36) and the proportion of mothers that practiced exclusive breastfeeding was 40.6%.  Married mothers were more likely to practice exclusive breastfeeding than their unmarried counterparts (AOR:6.324, 95%CI:1.809–22.114). The common reasons for not practicing exclusive breastfeeding were work schedule 57(26.3%), cultural beliefs and the need to introduce herbal medicine 32(14.7%), and insufficient breast milk 30(13.8%).  Conclusion: This study revealed a good disposition with a suboptimal practice towards exclusive breastfeeding. Also, being married was a positive predictor of exclusive breastfeeding. Therefore, we recommend policies that will improve exclusive breastfeeding among mothers in rural areas, especially those targeting the unmarried, in order to achieve the World Health Organization’s target. |
| Order of Authors: | Tope Michael Ipinnimo, MBBS, MPH, FWACP, FMCPH |
|  | Olanrewaju Kassim Olasehinde, MBBS, MWACP |
|  | Taofeek Adedayo Sanni, MBBS, MPH, PhD, FWACP, FMCPH |
|  | Ayodeji Andrew Omotoso, MBBS |
|  | Rita Omobosola Alabi, MBBS |
|  | Paul Oladapo Ajayi, MBBS, MPH, FWACP |
|  | Kayode Rasaq Adewoye, MBBS, MPH, MBA, FWACP |
|  | John Olujide Ojo, MBBS, MPH, FWACP |
|  | Olayinka Oloruntoba, MBBS, MWACS |
|  | Blessing Omobolanle Osho |
|  | Adewumi Rufus Fajugbagbe |
|  | Precious Aderinsola Adeyeye |
|  |  |

|  | Ayotomiwa Fiyinfoluwa Ajayi |
| --- | --- |
| Response to Reviewers: | Dear Academic Editor and Reviewers,  Thank you for taking the time to read and review this work, we are grateful for your insightful comments and contributions. Please find the response to each point raised within the uploaded rebuttal letter. |
| Additional Information: |  |
| Question | Response |
| Financial Disclosure  Enter a financial disclosure statement that describes the sources of funding for the work included in this submission. Review the [submission guidelines](http://journals.plos.org/plosone/s/submission-guidelines#loc-financial-disclosure-statement) for detailed requirements. View published research articles from [*PLOS ONE*](https://journals.plos.org/plosone/) for specific examples.  This statement is required for submission and will appear in the published article if the submission is accepted. Please make sure it is accurate. | The author(s) received no specific funding for this work. |
| Funded studies  Enter a statement with the following details:   - Initials of the authors who received each award - Grant numbers awarded to each author - The full name of each funder - URL of each funder website - Did the sponsors or funders play any role in the study design, data collection and analysis, decision to publish, or preparation of the manuscript? |  |
| Did you receive funding for this work? |  |
| Competing Interests  Use the instructions below to enter a competing interest statement for this submission. On behalf of all authors, disclose any [competing interests](http://journals.plos.org/plosone/s/competing-interests) that could be perceived to bias this work—acknowledging all financial support and any other relevant financial or non- financial competing interests. | The authors have declared that no competing interests exist |

| This statement is required for submission and will appear in the published article if the submission is accepted. Please make sure it is accurate and that any funding sources listed in your Funding Information later in the submission form are also declared in your Financial Disclosure statement.  View published research articles from  [*PLOS ONE*](http://journals.plos.org/plosone/) for specific examples. |  |
| --- | --- |
| NO authors have competing interests  Enter: *The authors have declared that no competing interests exist.*  Authors with competing interests  Enter competing interest details beginning with this statement:  *I have read the journal's policy and the authors of this manuscript have the following competing interests: [insert competing interests here]* |  |
| * typeset |  |
| Ethics Statement  Enter an ethics statement for this submission. This statement is required if the study involved:   - Human participants - Human specimens or tissue - Vertebrate animals or cephalopods - Vertebrate embryos or tissues - Field research   Write "N/A" if the submission does not require an ethics statement.  General guidance is provided below. Consult the [submission guidelines](http://journals.plos.org/plosone/s/submission-guidelines#loc-guidelines-for-specific-study-types) for detailed instructions. Make sure that all information entered here is included in the Methods section of the manuscript. | Ethical approval (ERC/2021/10/18/937A) for this study was obtained from the Human Research and Ethics Review Committee of the Federal Teaching Hospital, Ido-Ekiti, Nigeria. Permission was obtained from the officer-in-charge of the BHC and CHC as well as the consultant-in-charge of the FTH clinics. Additionally, written informed consent to participate was obtained from the mothers prior to the interviews. |

| Format for specific study types  Human Subject Research (involving human participants and/or tissue)   - Give the name of the institutional review board or ethics committee that approved the study - Include the approval number and/or a statement indicating approval of this research - Indicate the form of consent obtained (written/oral) or the reason that consent was not obtained (e.g. the data were analyzed anonymously)   Animal Research (involving vertebrate animals, embryos or tissues)   - Provide the name of the Institutional Animal Care and Use Committee (IACUC) or other relevant ethics board that reviewed the study protocol, and indicate whether they approved this research or granted a formal waiver of ethical approval - Include an approval number if one was obtained - If the study involved *non-human primates*, add *additional details* about animal welfare and steps taken to ameliorate suffering - If anesthesia, euthanasia, or any kind of animal sacrifice is part of the study, include briefly which substances and/or methods were applied   Field Research  Include the following details if this study involves the collection of plant, animal, or other materials from a natural setting:   - Field permit number - Name of the institution or relevant body that granted permission |  |
| --- | --- |
|  |  |
| Data Availability  Authors are required to make all data underlying the findings described fully available, without restriction, and from the time of publication. PLOS allows rare exceptions to address legal and ethical concerns. See the [PLOS Data Policy](http://journals.plos.org/plosone/s/data-availability) and [FAQ](http://journals.plos.org/plosone/s/data-availability#loc-faqs-for-data-policy) for detailed information. | Yes - all data are fully available without restriction |

| A Data Availability Statement describing where the data can be found is required at submission. Your answers to this question constitute the Data Availability Statement and will be published in the article, if accepted. |  |
| --- | --- |
| Important: Stating ‘data available on request from the author’ is not sufficient. If your data are only available upon request, select ‘No’ for the first question and explain your exceptional situation in the text box. |  |
| Do the authors confirm that all data underlying the findings described in their manuscript are fully available without restriction? |  |
| Describe where the data may be found in full sentences. If you are copying our sample text, replace any instances of XXX with the appropriate details.   - If the data are held or will be held in a public repository, include URLs, accession numbers or DOIs. If this information will only be available after acceptance, indicate this by ticking the box below. For example: *All XXX files are available from the XXX database (accession number(s) XXX, XXX.*). - If the data are all contained within the manuscript and/or Supporting Information files, enter the following: *All relevant data are within the manuscript and its Supporting Information files.* - If neither of these applies but you are able to provide details of access elsewhere, with or without limitations, please do so. For example:   *Data cannot be shared publicly because of [XXX]. Data are available from the XXX Institutional Data Access / Ethics Committee (contact via XXX) for researchers who meet the criteria for access to confidential data.*  *The data underlying the results presented in the study are available* | All relevant data are within the manuscript and its Supporting Information files |

| *from (include the name of the third party and contact information or URL).*   - This text is appropriate if the data are owned by a third party and authors do not have permission to share the data.   * typeset |  |
| --- | --- |
| Additional data availability information: |  |

<https://www.editorialmanager.com/pone/download.aspx?id=35248396&guid=1005aa6f-5522-4d79-a217-a473574a9f50&scheme=1>Manuscript	[Click here to access/download;Manuscript;Manuscript - Breatfeeding in Ido-Ekiti.docx](https://www.editorialmanager.com/pone/download.aspx?id=35248396&guid=1005aa6f-5522-4d79-a217-a473574a9f50&scheme=1)

# Title Page

**Title:** Attitude and Predictors of Exclusive Breastfeeding Practice among Mothers Attending Under-Five Welfare Clinics in a Rural Community in Southwestern Nigeria

# Contributors and their affiliation:

1. Tope Michael Ipinnimo

Department of Community Medicine, Federal Teaching Hospital, Ido-Ekiti, Nigeria.

2. Olanrewaju Kassim Olasehinde

Department of Community Medicine, Federal Teaching Hospital, Ido-Ekiti, Nigeria.

3. Taofeek Adedayo Sanni

Department of Community Medicine, Federal Teaching Hospital, Ido-Ekiti, and Afe Babalola University, Ado-Ekiti, Nigeria.

4. Ayodeji Andrew Omotoso

Department of Community Medicine, Federal Teaching Hospital, Ido-Ekiti, Nigeria.

5. Rita Omobosola Alabi

Department of Community Medicine, Federal Teaching Hospital, Ido-Ekiti, Nigeria.

6. Paul Oladapo Ajayi

Department of Community Medicine, Ekiti State University, Ado-Ekiti, Nigeria.

7. Kayode Rasaq Adewoye

Department of Community Medicine, Federal Teaching Hospital, Ido-Ekiti, and Afe Babalola University, Ado-Ekiti, Nigeria.

8. John Olujide Ojo

Department of Community Medicine, Federal Teaching Hospital, Ido-Ekiti, and Afe Babalola University, Ado-Ekiti, Nigeria.

9. Olayinka Oloruntoba

Department of Surgery, Federal Teaching Hospital, Ido-Ekiti, Nigeria.

10. Blessing Omobolanle Osho

Department of Public Health, Kwara State University, Malete, Nigeria.

11. Adewumi Rufus Fajugbagbe

Department of Public Health, Kwara State University, Malete, Nigeria.

12. Precious Aderinsola Adeyeye

Department of Public Health, Kwara State University, Malete, Nigeria.

13. Ayotomiwa Fiyinfoluwa Ajayi

Department of Public Health, Kwara State University, Malete, Nigeria.

# Corresponding Author:

Name: Tope Michael Ipinnimo

Address: Department of Community Medicine, Federal Teaching Hospital, P.M.B 201, Ido-Ekiti, Nigeria.

Phone numbers: +2348062201903

E-mail address: [abbeymagnus@yahoo.com](mailto:abbeymagnus@yahoo.com)

# Ethical approval and consent to participate:

Ethical approval for the study was sought and obtained from the Ethics and Research Review Committee of the Federal Teaching Hospital, Ido-Ekiti, Nigeria. Informed consent was obtained from all the research subjects.

# Competing Interest:

The Authors declare they have no competing interest in conducting this study.

# Funding:

None

# Contribution details:

Concept and design- TMI, OKO, TAS Literature search- All Authors

Data collection- All Authors Data analysis- TMI, OKO

Manuscript Preparation- TMI, AAO, ROA, POA Manuscript Editing- All Author

Manuscript Review- All Author

All authors read and approved the final draft of the manuscript.

# Acknowledgment:

We express gratitude to all the participants of this study.

# Attitude and Predictors of Exclusive Breastfeeding Practice among Mothers Attending Under-Five Welfare Clinics in a Rural Community in Southwestern Nigeria

**Abstract**

***Background:*** Much previous research on exclusive breastfeeding has focused on urban and semi-urban communities, while there is still a paucity of data from the rural areas. We assessed the attitude and practice of exclusive breastfeeding and its predictors among mothers attending the under-five welfare clinics in a rural community.

***Methods:*** A cross-sectional study was conducted among consecutively recruited 217 mothers attending the three health facilities under-five welfare clinics in Ido-Ekiti, Southwest, Nigeria. Information was collected with a semi-structured interviewer-administered questionnaireadapted from previously published research works. Descriptive and inferential statistics were carried out with the aid of IBM SPSS Statistics for Windows, Version 26.0.

***Results:*** More than half of the mothers, 117(53.9%) were ≥30 years, and 191(88.0%) were married. Almost all 216 (99.5%) attended an ante-natal clinic and 216 (99.5%) attended an ante-natal clinic; however, 174(80.2%) delivered in the health facility. The respondent’s mean ± SD exclusive breastfeeding attitudinal score was

29.94 ± 2.14 (maximum obtainable score was 36), and the proportion of mothers that practiced exclusive breastfeeding was 40.6%. Married mothers were more likely to practice exclusive breastfeeding than their unmarried counterparts (AOR:6.324, 95%CI:1.809–22.114). The common reasons for not practicing exclusive breastfeeding were work schedule 57(26.3%), cultural beliefs and the need to introduce herbal medicine 32(14.7%), and insufficient breast milk 30(13.8%).

***Conclusion:*** This study revealed a good disposition with a suboptimal practice towards exclusive breastfeeding. Also, being married was a positive predictor of exclusive breastfeeding. Therefore, we recommend policies that will improve exclusive breastfeeding among mothers in rural areas, especially those targeting the unmarried, in order to achieve the World Health Organization’s target.

***Keywords:*** Attitude, exclusive breastfeeding, practice, Nigeria, rural

# Introduction

Breastmilk is described as the primary source of nutrition for newborns and provides all the nutritional needs for the first few months of life. [1] It is safe, clean and essentially contains water, nutrients, and antibodies in adequate proportions to promote child growth and development. [2] Exclusive breastfeeding (EBF) involves feeding only breastmilk to a child for the first six months of life, except for medically prescribed drugs or supplements. [3] The World Health Organisation (WHO) recommends EBF because it is cost-effective, and significantly

lower the risk of diarrhoea, malnutrition as well as morbidity and mortality among the under-five age group. [2] Breastfeeding not only proves beneficial to child health but also contributes to maternal health as well as providing social and economic benefits. [4]

Globally, the average EBF practice between 2015-2021 was 48% [5] and in Italy, only 33.3% practiced EBF. More than half of the Italian women had heard about EBF, with the majority of them believing that EBF is important to both mother and child. [6] However, a study in Bangladesh, which focused on mothers in rural areas, showed poor knowledge (34.5%) and practice (27.9%) of EBF. [7] In East Africa, a systematic review revealed that 42% of mothers preferred to feed their infants with breastmilk alone for the first six months of life, while 55.9% of them had practised EBF for at least six months. [8] The West Africa region had a prevalence of 35.0% for EBF between 2015-2021, one of the lowest in the world for the same period. [5]

In Nigeria, only 28.7% of babies were exclusively breastfed in 2018. [9] This was even lower in the rural population, with a report of 24.3%. [9] A study in a semi-urban area of Sokoto state, Northwest Nigeria, indicated that only 31.0% of women practiced EBF. [10] Another study conducted in one of the urban centers in Abuja, the Federal Capital Territory of Nigeria showed that 54.4% had practiced EBF despite a significant positive attitude determined by the study (70% agreed that EBF was adequate for their children). [11] A similar study in another urban center in Benin, Edo state, South-south Nigeria, showed an “ever-breastfed rate” of 100.0%,but only 40.7% practiced EBF. [12] A survey carried out in two tertiary hospitals in Ogun state, Southwest Nigeria, revealed that more than half (58.8%) of the women practiced EBF. [13]

Optimal breastfeeding practices are important in of preventing and managing malnutrition in children. [2] Stunting, which is a form of malnutrition and an indication of poor nutrition beginning in utero into early childhood, affecting an estimated 144 million (21.3%) under-five children globally in 2019 with the highest burdens in Africa and Asia continents. [14] Over one-third (37.0%) of Nigerian children aged 6 - 59 months are stunted, and the prevalence of stunting is almost twice as high among children in rural areas (45.0%) as among those in urban areas (27.0%). [9]

Many previous studies on EBF have centered on urban and semi-urban communities, while there is still a paucity of data from rural areas. [10-13] To the best of the authors’ knowledge, there has not been a study of this nature assessing the attitude and practice of mothers on EBF in our environment. This research was designed to identify the gap in the attitude and practice of mothers living in this area toward EBF as it may aid strategy development in improving the nutritional status of infants and children living in the communities.

This study aims to determine the attitude and practice and identify the predictors of practice of EBF among mothers attending the under-five welfare clinics in a rural community in Southwest Nigeria. The findings of this work would contribute to the literature and can help facilitate further research on the subject. Also, it would aid in defining the gaps in women’s attitude to EBF and its practice, especially in the rural areas. This could then serve as a basis for health planning, policy formulation and implementation to improve EBF practice in rural Nigeria in

order to meet the World Health Organization (WHO) recommended target of at least 50.0% EBF rate in all communities by 2025. [15]

# Methods

## Study area and design

This was a cross-sectional study carried out between 1st and 30th November 2021 in all the under-five welfare clinics in Ido-Ekiti, Southwest, Nigeria. Ido-Ekiti is a town with an estimated population of 37,000. [16] There are three health facilities with under-five welfare clinic in the community which included the Basic Health Centre (BHC), Comprehensive Health Centre (CHC) and the Federal Teaching Hospital (FTH), Ido-Ekiti. The BHC and CHC are primary health facilities, each with a staff strength of less than thirty healthcare workers mainly registered nurses/midwives and community health extension workers. The BHC and CHC under-five welfare clinics provides services such as immunization and growth monitoring on clinic days which is just once in a week and each of them sees an average of 10 to 20 children per clinic day. The FTH is a tertiary health facility that serves as a referral centre for other health facilities within the environs. The under-five welfare clinic in FTH is located at the Department of Community Medicine of the hospital and it offers services every day with the exception of weekends (Saturday to Sunday) to an average of 20 children per day. The staff in the clinic consists of community/public health physicians, public health nurses and community health extension workers.

## Participants, sample size determination and sampling technique

The study population consisted of mothers attending the under-five welfare clinics in the community. The study included all healthy mothers with healthy baby(ies) attending the clinics. Mothers with adopted or fostered babies were excluded from the study. The minimum sample size for the study was determined using Leslie Fisher’s formula. [17] A 95% confidence interval, and 5% degree of accuracy was assumed. A sample size of 217 was obtained after using the prevalence of EBF from a previous study. [18] All the three health facilities were used for the study. The number of mothers selected from the health facilities was determined using proportionate allocation based on the under-five welfare clinic attendance. Eligible mothers attending the clinic were recruited consecutively into the study until the sample size was achieved.

## Data Collection Methods

Researchers administered the questionnaire in a scheduled area of the health facilities through face-to-face interviews with the mothers immediately after their clinics.

## Data Collection Tools

The study instrument used to collect information from the women is a 26-item semi-structured interviewer-administered questionnaire adapted from previously published research works. [19, 20] The questionnaire obtained data on socio-demographic and other (such as antenatal clinic

attendance, place of delivery) variables, attitude towards EBF, practice of EBF and reasons for not breastfeeding exclusively. The questionnaire was pre-tested on a sample (twenty) of mothers in a CHC in another community which were neither analyzed nor included in the study. Necessary adjustments and corrections were made to the questionnaire after the pre-test. The items in the study instrument were tested for internal consistency using the Cronbach coefficient alpha test and a score of 0.9 was obtained. Twelve questions assessed the attitude of mothers on EBF. Mothers were asked to rate their responses on a 3-point Likert scale (1 to 3) measuring the intensity of mother’s attitudes. [19] A positive response was rated 3, a neutral was rated 2, and a negative was rated 1. The attitudinal score of each respondent was estimated by summing their rating with a maximum obtainable score of 36. Exclusive breastfeeding practice was assessed with a question inquiring if mothers have given only breast milk to the baby for the first 6 months without infant formula or any other product unless it was prescribed by a physician.

## Variables

The independent variables in this study were sociodemographic variables, maternal characteristics such as antenatal clinic attendance, place of delivery and mode of delivery, as well as attitude towards EBF while the dependent variable included the practice of EBF among the mothers.

## Data management and statistical analysis

The data collected were entered, cleaned and analysed using computer software IBM SPSS (IBM SPSS Statistics for Windows, Version 26.0. Armonk, NY: IBM Corp). Data were summarized in tables using frequencies and percentages. The attitudinal scores of the participants were calculated and presented using mean and standard deviation. The mean attitudinal scores across each socio-demographic and maternal variables category was compared using the independent Student-T-test. The association between the mother’s characteristics and the practice of EBF was assessed using the Chi-square test. Binary logistic regression analysis was used to determine the predictors of EBF. The logistic regression model was built with variables with p ≤ 0.2 at the bivariate (Chi-square test) level of analysis. P-values <0.05 were considered significant for the inferential statistics.

## Ethical Considerations

Ethical approval (ERC/2021/10/18/937A) for this study was obtained from the Human Research and Ethics Review Committee of the Federal Teaching Hospital, Ido-Ekiti, Nigeria. Permission was obtained from the officer-in-charge of the BHC and CHC as well as the consultant-in-charge of the FTH clinics. Additionally, written informed consent to participate was obtained from the mothers before the interviews. Confidentiality and anonymity were maintained by not collecting personal data such as names and phone numbers from the respondents.

# Results

Table 1 shows that more than half of the mothers, 117 (53.9%), were ≥ 30 years and more than two-thirds, 148 (68.2%),held a post-secondary school degree. More than three-quarters, 191 (88.0%) and 179 (82.5%) were married and of Yoruba ethnicity respectively. Ninety-four (43.3%) of them were civil servants. The majority 216 (99.5%) of mothers attended the ante-

natal clinic, out of which 214 (99.1%) were informed on EBF. Most, 174 (80.2%) delivered within a health facility and 182 (83.9%) deliveries were via vaginal delivery. Ninety-one (41.9%) mothers are currently nursing their first child.

Table 2 shows that the mean (standard deviation) EBF attitudinal score for all respondents was

29.94 (2.14) out of 36 maximum possible scores. EBF attitudinal scores significantly differed across age (T = -2.844, p = 0.005), level of education (T = -2.273, p = 0.025), ante-natal clinic attendance during the pregnancy of the baby (T = 4.895, p < 0.001), been informed on EBF during ante-natal clinic (T = 14.378, p < 0.001), mode of delivery (T = -2.773, p = 0.008) and the birth order of the child (F = 9.533, p = 0.001). The attitudinal score was better among mothers aged ≥ 30 years, those with post-secondary education, mothers with antenatal clinic attendance, those informed on EBF, mothers who had caesarian section delivery, and those with higher birth orders. The attitudinal score did not significantly differ across other variables.

Eighty-eight (40.6%) mothers practised EBF,had EBF, and there was a statistically significant association between marital status and the practice of EBF (X = 8.993, p = 0.003). A higher proportion of married mothers practised EBF than unmarried mothers (married: 44.5%; unmarried: 11.5%). There was no statistically significant association between other respondents’ variables and the practice of EBF. Binary logistic regression in Table 4 revealed that married mothers were about 6 times more likely to practice EBF than those who were unmarried (AOR:6.324, 95%CI: 1.809 – 22.114) (Table number).

According to Figure 1, the reasons for the mothers not practicing EBF were work schedule 57 (26.3%), cultural beliefs and the need to introduce herbal medicine 32 (14.7%), insufficient breast milk 30 (13.8%), baby getting hungry and thirsty 28 (12.9%), HIV and unplanned pregnancy 19 (8.8%), early and single motherhood 14 (6.5%) and other reasons 4 (1.8%).

# Discussion

This study assessed the attitude towards EBF, its practice, and predictors among mothers attending under-five welfare clinics in a rural community. The mean attitude score towards EBF for all mothers in this study was 29.9 out of 36 maximum possible scores. This is 83.2% of the maximum obtainable score which depicts a good disposition towards EBF among the mothers. This finding is higher than what was obtained in rural communities of Lagos, Nigeria. [20] However, it is similar to what was found in studies done among lactating mothers in a rural Ghana community and Ethiopia. [19, 21] This level of attitude is good for the mothers, although there is room for improvement.

This study revealed that EBF attitude was positively associated with age, as mothers who were 30 years and over had a better attitude than those aged less than 30 years. This finding is consistent with findings from studies in Ondo state, Southwest, Nigeria [22] and in the Island of Abu Dhabi, United Arab Emirates. [23] Also, mothers who had attended the antenatal clinic during the pregnancy of the baby had a better attitude than mothers who did not attend the clinic. The antenatal clinic provides nutritional counseling and education including the health benefits of EBF and other infant feeding practices as part of the health promotion measures during visits.

Unsurprisingly, this exposure not only improves maternal attitude but subsequently brings about the likelihood of better practice as reported in a study done in Rawalpindi. [24]

This study found that mothers with a higher level of education had better dispositions toward EBF. Maternal level of education is a significant determinant of infant feeding practices, and this is supported by a study done in Ondo state, Southwest, Nigeria which found that attitude towards EBF is higher among lactating mothers with higher levels of education. [22] Mothers with higher levels of education can understand and quickly realize the benefits of EBF to their infants and are more motivated to practice it. Programs lower promoting EBF should be adapted to appeal to mothers with lower education levels of education education. The maternal attitude of EBF was better among mothers with 2^nd^ birth than those with their first birth. This is similar to the findings in a study done in Ibadan, Southwest, Nigeria. [25] This finding is not surprising as multiparous mothers have previously developed a prior experience and skillset, allowing for better EBF attitude in subsequent deliveries.

Less than half (40.6%) of mothers in this study practiced EBF. This is similar to a study done in Edo state, South-south, Nigeria with a prevalence of 40.7%. [12] However, it is higher than the findings in previous studies done in Italy (33.3%), Bangladesh (34.5%), Southern Ethiopia (14.9%), Jigawa (26.8%) and Sokoto (31.0%) states, Northern, Nigeria (31.0%) [6, 7, 10, 26, 27] and lower than the prevalence recorded from studies conducted in Debre Berhan District, Ethiopia (68.6%), urban centers of Abuja (54.4%), Ikorodu, Lagos (69.4%) and in Ogun State, Southwest, Nigeria (58.8%). [11, 13, 28, 20] Interestingly, the prevalence of practice of EBF in this study aligns with the WHO targets of at least 50.0% rate in all communities whether rural or urban by 2025. [15] Though, close to the WHO target, several barriers still hinder the practice of EBF. Work schedule, cultural beliefs and the need to introduce herbal medicine, insufficient breast milk, baby gets hungry and thirsty, HIV and unplanned pregnancy, early and single motherhood were mentioned by the mothers as barriers to EBF. Some of these barriers have equally been reported in previous studies. [29, 30]

It was observed from this study that mothers who are married were about 6 times more likely to practice EBF than those who are unmarried. Expectedly, this result is substantiated by another study in Ethiopia. [31] This may be due to the responsibility-sharing and support provided by the husband that could aid the practice of EBF. However, this finding contrast that found in a study done among first-time mothers in Ethiopia which revealed that participants who were not married were almost 3-fold more likely to practice EBF compared with married counterpart. [32] This difference could be due to birth-related traditional practices of some Ethiopian where first- time mothers at 8 months gestational age visit their parent’s home in preparation for births, and afterward, stay for 40 days after birth with their infant and never left alone. Against these background, one can deduce that a good support system from husbands of married women or from parents of unmarried women post-delivery makes the practice of EBF more feasible.

# Strengths and limitations

To the best of our knowledge, this is the first study in Ekiti state that assessed the attitude and practice of exclusive breastfeeding among rural mothers. We also identified predictors of exclusive breastfeeding among this population.

However, the study shares the limitations of the cross-sectional study design. In addition, the research is prone to social desirability and recall bias since questions were asked about the past experiences of the mothers on breastfeeding. Lastly, this health facility-based study was carried out in the under-five welfare clinic and might have left out mothers who do not seek healthcare or patronize other institutions such as patent medicine stores, drug hawkers and herbalists for their healthcare.

# Conclusion and recommendation

The study revealed a good disposition towards EBF among mothers with less than half (40.6%) breastfeeding their babies exclusively. The common reasons given for not breastfeeding exclusively included work schedule, cultural beliefs, and the need to introduce herbal medicine, insufficient breast milk and the baby gets hungry and thirsty. Also, it was found that married mothers were more likely to practice EBF than unmarried mothers. We, therefore, recommend policies that will improve the EBF of mothers in rural areas toward achieving the WHO target. It is also important to carry out further quantitative and qualitative studies to identify factors associated with low uptake of EBF among unmarried women and develop interventions to improve their practice.

# Key messages

- The global average of exclusive breastfeeding practice between 2015-2021 was 48%, while the West Africa region had a prevalence of 35.0%, which is 15% less than the World Health Organization’s recommended target of at least 50.0% in all communities by the year 2025.
- This prevalence is even lower among the rural population, where the least research that could affect policy and practice on exclusive breastfeeding has been carried out.
- This study which was done in a rural community found that the attitude of the mothers was good, however, the practice of exclusive breastfeeding was still suboptimal. In addition, being married was a positive predictor of exclusive breastfeeding among this population.
- Policies that will improve exclusive breastfeeding among mothers in rural areas were recommended. Furthermore, in-depth research into the factors implicated in the low uptake of exclusive breastfeeding among unmarried women is equally advocated.

# Competing Interest

Authors declare they have no competing interest in conducting this study

# Supporting information

S1 File. Minimal data set.

S2 File. Ethics committee approval.

# References

1. World Health Organization. Breastfeeding. 2024. Available from: [https://www.who.int/health-topics/breastfeeding#tab=tab_1](https://www.who.int/health-topics/breastfeeding#tab%3Dtab_1) [Last accessed 12 January 2024]
2. World Health Organization. Infant and young child feeding. 2021. Available from: <https://www.who.int/news-room/fact-sheets/detail/infant-and-young-child-feeding> [Last accessed 8 February 2023]
3. Centre for Disease Control and Prevention. Nutrition: Infant and Toddler nutrition. 2022. <https://www.cdc.gov/nutrition/infantandtoddlernutrition/definitions.html> [Last accessed 8 February 2023]
4. The Innocenti declaration on the protection, promotion, and support of breastfeeding. 1990. Florence, Italy. World Health Organization/United Nation Children’s Fund.
5. United Nation Children’s Fund. Global Database for Exclusive Breastfeeding. 2022. Available from: [https://data.unicef.org/topic/nutrition/breastfeeding](https://data.unicef.org/topic/nutrition/breastfeeding/)/ [Last accessed 8 February 2023]
6. Cascone D, Tomassoni D, Napolitano F, Di Giuseppe G. Evaluation of Knowledge, Attitudes, and Practices about Exclusive Breastfeeding among Women in Italy. Int J Environ Res Public Health. 2019;16(12):2118. DOI: 10.3390/ijerph16122118.
7. Rana MM, Islam MR, Karim MR, Islam AZ, Haque MA, Shahiduzzaman M, Hossain MG. Knowledge and practices of exclusive breastfeeding among mothers in rural areas of Rajshahi district in Bangladesh: A community clinic-based study. PLoS One. 2020;15(5):e0232027. DOI: 10.1371/journal.pone.0232027.
8. Dukuzumuremyi JPC, Acheampong K, Abesig J, Luo J. Knowledge, attitude, and practice of exclusive breastfeeding among mothers in East Africa: a systematic review. Int Breastfeed J. 2020;15(1):70. DOI: 10.1186/s13006-020-00313-9.
9. National Demographic and Health Survey. 2018. National Population Commission. Federal Republic of Nigeria.
10. Oche MO, Umar AS, Ahmed H. Knowledge and practice of exclusive breastfeeding in Kware, Nigeria. African Health Sciences 2011; 11(3): 518 – 523
11. Okoroiwu GIA, Ubosi NI, Aliyu SM, Eya CP. Knowledge, Attitude and Practice of Exclusive Breastfeeding amongst Mothers of Infants in Gwagwalada Area Council, FCT,

Abuja,	Nigeria.	J.	Appl.	Sci.	Environ.	Manage.	2021;25(1):127-132. <https://dx.doi.org/10.4314/jasem.v25i1.18>

12. Akpan UJ, Ibadin MO, Abiodun PO. Breastfeeding practices in early infancy in Benin City, Nigeria. Niger J Paed. 2015;42(2): 126 –131. <http://dx.doi.org/10.4314/njp.v42i2.11>
13. Adebayo A, Oluwaseyi O. Breastfeeding Practices among Mothers in Southwest Nigeria. Ethiop J Health Sci. 2020;30(5):697. <http://dx.doi.org/> 10.4314/ejhs.v30i5.8
14. United Nations Children's Fund, World Health Organization & World Bank. 2020. Levels and trends in child malnutrition: UNICEF/WHO/The World Bank Group joint child malnutrition estimates: key findings of the 2020 edition. World Health Organization. Available from: <https://apps.who.int/iris/handle/10665/331621> [Last accessed 4 March 2023]
15. World Health Organization. Global nutrition targets 2025: Policy brief series. 2014. Available from: <https://www.who.int/publications/i/item/WHO-NMH-NHD-14.2> [Last accessed 4 March 2023]
16. City-facts. Ido-Ekiti. 2015. Available from: <https://www.city-facts.com/ido-ekiti> [Last accessed 4 March 2023]
17. Ipinnimo TM, Abodunrin OL, Durowade KA. Relationship between Cost of Care and Medication Adherence among Hypertensive Patients attending a Tertiary Health Facility in Ekiti State, Nigeria. Journal of community medicine and primary health care. 2022; 34(1):38-57. <https://dx.doi.org/10.4314/jcmphc.v34i1.3>
18. Adewuyi EO, Adefemi K. Breastfeeding in Nigeria: a systematic review. Int J Community Med Public Health. 2016;3(2):385-396. [http://dx.doi.org/10.18203/2394-](http://dx.doi.org/10.18203/2394-6040.ijcmph20160421) [6040.ijcmph20160421](http://dx.doi.org/10.18203/2394-6040.ijcmph20160421)
19. Mogre, V., Dery, M., Gaa, P.K. Knowledge, attitudes and determinants of exclusive breastfeeding practice among Ghanaian rural lactating mothers. Int Breastfeed J. 2016;11:12. <https://doi.org/10.1186/s13006-016-0071-z>
20. Balogun MR, Okpalugo OA, Ogunyemi AO, Sekoni AO. Knowledge, Attitude, and Practice of Breastfeeding: A Comparative Study of Mothers in Urban and Rural Communities of Lagos, Southwest Nigeria. Niger Med J. 2017;58(4):123-130. doi: 10.4103/nmj.NMJ_289_16.
21. Tadele N, Habta F, Akmel D, Deges E. Knowledge, attitude and practice towards exclusive breastfeeding among lactating mothers in Mizan Aman town, Southwestern Ethiopia: descriptive cross-sectional study. Int Breastfeed J. 2016;11:3. DOI: 10.1186/s13006-016-0062-0.
22. Adegboro JS. Influence of demographic variables on attitude of nursing mothers towards exclusive breastfeeding in Akoko South West Local Government Area of Ondo State,

Nigeria. International Journal of Life Science Research Archive. 2022;3(1):031-8. <https://doi.org/10.53771/ijlsra.2022.3.1.0065>

23. Al Ketbi MI, Al Noman S, Al Ali A, Darwish E, Al Fahim M, Rajah J. Knowledge, attitudes, and practices of breastfeeding among women visiting primary healthcare clinics on the island of Abu Dhabi, United Arab Emirates. Int Breastfeed J. 2018;13:26. DOI: 10.1186/s13006-018-0165-x.
24. Ahmad MO, Sughra U, Kalsoom U, Imran M, Hadi U. Effect of antenatal counseling on exclusive breastfeeding. J Ayub Med Coll Abbottabad. 2012;24(2):116-9.
25. Ayinde AO, Adedokun B, Adeloye AI, Agbonjimi LA, Ayinde TO, Ogunode NJ. Birth order and exclusive breastfeeding practices among mothers with two or more children. Ijcsmr. 2021;1(1):1-10. <https://journalofmedical.org/index.php/ijcsmr>
26. Ermancho B., Lette A., Tamirat A., Ermeko T. Determinants of exclusive breastfeeding practice in Southern Ethiopia. J. Food Nutr. Popul. Health. 2021;5(4):50.
27. Sabo A, Abba J, Sunusi Usman U, Musa Saulawa I, Alzoubi MM, Al-Mugheed K, Alsenany SA, Farghaly Abdelaliem SM. Knowledge, attitude, and practice of exclusive breastfeeding among mothers of childbearing age. Front Public Health. 2023;11:1277813. doi: 10.3389/fpubh.2023.1277813.
28. Asfaw MM, Argaw MD, Kefene ZK. Factors associated with exclusive breastfeeding practices in Debre Berhan District, Central Ethiopia: a cross sectional community based study. Int Breastfeed J. 2015 Aug 13;10:23. doi: 10.1186/s13006-015-0049-2.
29. Otoo GE, Lartey AA, Pérez-Escamilla R. Perceived incentives and barriers to exclusive breastfeeding among periurban Ghanaian women. Journal of Human Lactation. 2009;25(1):34-41. doi: 10.1177/0890334408325072.
30. Apanga PA. A review on facilitators and barriers to exclusive breastfeeding in West Africa. Journal of Biology, Agriculture and Healthcare. 2014;4:9–15.
31. Mamo K, Dengia T, Abubeker A, Girmaye E. Assessment of Exclusive Breastfeeding Practice and Associated Factors among Mothers in West Shoa Zone, Oromia, Ethiopia. Obstet Gynecol Int. 2020;2020:3965873. doi: 10.1155/2020/3965873.
32. Ayalew T. Exclusive breastfeeding practice and associated factors among first-time mothers in Bahir Dar city, North West Ethiopia, removed: A community based cross sectional study. Heliyon. 2020;6(9):e04732. doi:10.1016/j.heliyon.2020.e04732

# Tables

**Table 1** Socio-demographic and other characteristics of the respondents (N=217)

| **Variable** | **Frequency (N= 217)** | **Percent (%)** |
| --- | --- | --- |
| **Age**  < 30 years | 100 | 46.1 |
| ≥ 30 years | 117 | 53.9 |
| **Level of education**  Secondary | 69 | 31.8 |
| Post-secondary | 148 | 68.2 |
| **Marital status**  Unmarried | 26 | 12.0 |
| Married | 191 | 88.0 |
| **Religion**  Christianity | 174 | 80.2 |
| Islam | 43 | 19.8 |
| **Tribe**  Yoruba | 179 | 82.5 |
| Hausa | 16 | 7.4 |
| Igbo | 22 | 10.1 |
| **Occupation**  Farmer/trader/artisan | 76 | 35.0 |
| Civil servant | 94 | 43.3 |
| Professional | 20 | 9.2 |
| Student | 27 | 12.5 |
| **ANC attendance during the pregnancy of this baby**  Yes | 216 | 99.5 |
| No | 1 | 0.5 |
| **If yes, were you informed on exclusive breastfeeding (n=216)**  Yes | 214 | 99.1 |
| No | 2 | 0.9 |
| **Place of delivery of this baby**  Outside health facility | 43 | 19.8 |
| Within health facility | 174 | 80.2 |
| **Mode of delivery of this baby**  Vaginal delivery | 182 | 83.9 |
| Caesarean section | 35 | 16.1 |
| **Birth order of the baby**  First | 91 | 41.9 |
| Second | 75 | 34.6 |
| 3 and above | 51 | 23.5 |

**Table 2** Exclusive breastfeeding attitudinal score associated with respondents’ characteristics (N=217)

| **Variable** | **Attitudinal score Mean ± SD** | **Test** | **p-value** |
| --- | --- | --- | --- |
| **All respondents** | 29.94 ± 2.14 | - | - |
| **Age (in years)**  < 30 years | 29.50 ± 1.956 | -2.844^T^ | **0.005** |
| ≥ 30 years  **Level of education** | 30.30 ± 2.226 | -2.273^T^ | **0.025** |
| Secondary | 29.43 ± 2.29 |  |  |
| Post-secondary  **Marital status** | 30.16 ±2.02 | -0.480^T^ | 0.634 |
| Unmarried | 29.76 ± 1.839 |  |  |
| Married  **Religion** | 29.95 ± 2.180 | 1.974^T^ | 0.053 |
| Christianity | 30.09 ± 2.007 |  |  |
| Islam  **Tribe** | 29.27 ± 2.529 | 0.317^F^ | 0.729 |
| Yoruba | 29.97 ± 2.075 |  |  |
| Hausa | 30.00 ± 1.591 |  |  |
| Igbo  **Occupation** | 29.59 ± 2.938 | 0.427^F^ | 0.734 |
| Farmer/trader/artisan | 29.72 ± 2.194 |  |  |
| C/S | 30.09 ± 2.374 |  |  |
| Professional | 30.00 ± 2.000 |  |  |
| Student  **ANC attendance during the pregnancy of this baby** | 29.92 ± 0.196 | 4.895^T^ | **<0.001** |
| Yes | 29.98 ± 2.034 |  |  |
| No  **If yes, were you informed on exclusive breastfeeding**  Yes | 20.00 ± 0.000  30.00 ± 2.034 | 14.378^T^ | **<0.001** |
| No  **Place of delivery** | 28.00 ± 0.000 | 0.571^T^ | 0.570 |
| Outside health facility | 30.11 ± 2.372 |  |  |
| Within health facility  **Mode of delivery** | 29.98 ± 2.083 | -2.773^T^ | **0.008** |
| Vaginal delivery | 29.78 ± 2.166 |  |  |
| Caesarean section  **Birth order** | 30.74 ± 1.820 | 9.533^F^ | **0.001** |
| First | 29.45 ± 2.088 |  |  |
| Second | 30.77 ± 1.713 |  |  |
| Third and above | 29.56 ± 2.443 |  |  |

SD: Standard deviation, ^T^: Student T test, ^F^: Analysis of variance

**Table 3** Association between respondents’ characteristics and practice of exclusive breastfeeding (N=217)

**Exclusive breastfeeding**

| **Variable** | **Yes (n=88) (%)** | **No (n=129) (%)** | **Test** | **p-value** |
| --- | --- | --- | --- | --- |
| **Age group (years)**  <30 years | 41 (41.0) | 59 (59.0) | 0.015^X^ | 1.000 |
| >30 years  **Level of education**  Secondary | 47 (40.2)  29 (42.0) | 70 (59.8)  40 (58.0) | 0.091^X^ | 0.769 |
| Post-secondary  **Marital status**  Unmarried | 59 (39.9)  3 (11.5) | 89 (60.1)  23 (88.5) | 8.993^Y^ | **0.003** |
| Married  **Religion**  Christianity | 85 (44.5)  72 (41.4) | 106 (55.5)  102 (58.6) | 0.249^X^ | 0.729 |
| Islam  **Tribe** | 16 (37.2) | 27 (52.8) | 3.506^F^ | 0.173 |
| Yoruba | 75 (49.1) | 104 (58.9) |  |  |
| Hausa | 3 (18.8) | 13 (81.3) |  |  |
| Igbo  **Occupation**  Farmer/trader/artisan | 10 (45.5)  32 (42.9) | 12 (54.5)  44 (57.1) | 2.257^X^ | 0.529 |
| C/S | 34 (36.2) | 60 (63.8) |  |  |
| Professional | 8 (40.0) | 12 (60.0) |  |  |
| Student  **ANC attendance during the** | 14 (51.9) | 13 (48.1) | <0.001^Y^ | 1.000 |
| **pregnancy of this baby** |  |  |  |  |
| Yes | 88 (40.7) | 128 (59.3) |  |  |
| No  **If yes to question 7, were you informed** | 0 (0.0) | 1 (100.0) | 0.207^Y^ | 0.649 |
| **on exclusive breastfeeding**  Yes | 88 (41.1) | 126 (58.9) |  |  |
| No  **Place of delivery of this baby** | 0 (0.0) | 2 (100.0) | 0.715^X^ | 0.488 |
| Outside HF | 15 (34.9) | 28 (65.1) |  |  |
| Within HF  **Mode of delivery**  Vaginal delivery | 73 (42.0)  74 (40.7) | 101 (58.0)  108 (59.3) | 0.005^X^ | 1.000 |
| Caesarean section  **Birth order** | 14 (40.0) | 21 (60.0) | 2.859^X^ | 0.245 |
| First | 32 (35.2) | 59 (54.8) |  |  |
| Second | 36 (48.0) | 39 (52.0) |  |  |
| Third and above  **Mean attitudinal score** | 20 (39.2)  30.09 ± 1.891 | 31 (60.8)  29.82 ± 2.295 | 0.883^T^ | 0.378 |

^X^: Pearson Chi-Square, ^Y^: Continuity correction, ^F^: Fisher’s Exact Test

**Table 4** Predictors of practice of excusive breastfeeding

# Predictors

**95% CI for AOR**

|  | **B** | **P-value** | **AOR** | **Lower** | **Upper** |
| --- | --- | --- | --- | --- | --- |
| **Marital Status (**Married) | 1.844 | **0.004** | 6.324 | 1.809 | 22.114 |
| **Tribe** (Hausa) | 0.357 | 0.459 | 1.429 | 0.556 | 3.674 |
| **Tribe** (Igbo) | 1.429 | 0.072 | 4.173 | 0.879 | 19.803 |

B: Intercept, AOR: Adjusted odd ratio, CI: Confidence interval

<https://www.editorialmanager.com/pone/download.aspx?id=35241168&guid=4e4fbfec-cfec-40e2-bb32-a58e9a8ce890&scheme=1>Figure	[Click here to access/download;Figure;Figure - Breastfeeding in Ido-Ekiti.docx](https://www.editorialmanager.com/pone/download.aspx?id=35241168&guid=4e4fbfec-cfec-40e2-bb32-a58e9a8ce890&scheme=1)

# Attitude and Predictors of Exclusive Breastfeeding Practice among Mothers Attending Under-Five Welfare Clinics in a Rural Community in Southwestern Nigeria

60

**Reasons for not practicing exclusive breastfeeding***

57

50

40

32

30

30

28

19

20

14

10

4

0

Work schedule Cultural belief

and the need to introduce herbal medicine

Breast milk

was insufficient

The baby gets

hungry and the child being thirsty

HIV and

unintended pregnancies

Early and

single motherhood

Others

*****Multiple response

**Figure 1** Reasons for not practicing exclusive breastfeeding (N=217)

S1 File Data set

[Click here to access/download](https://www.editorialmanager.com/pone/download.aspx?id=35248431&guid=c5b2cc51-d6ba-48ff-ab25-6f6940eeb1e8&scheme=1)

[Supporting Information](https://www.editorialmanager.com/pone/download.aspx?id=35248431&guid=c5b2cc51-d6ba-48ff-ab25-6f6940eeb1e8&scheme=1)

[Data set - Breastfeeding in Ido-Ekiti.sav](https://www.editorialmanager.com/pone/download.aspx?id=35248431&guid=c5b2cc51-d6ba-48ff-ab25-6f6940eeb1e8&scheme=1)

Revised Manuscript with Track Changes

#### Title Page

**Title:** Attitude and Predictors of Exclusive Breastfeeding Practice among Mothers Attending Under-Five Welfare Clinics in a Rural Community in Southwestern Nigeria

#### Contributors and their affiliation:

1. Tope Michael Ipinnimo

Department of Community Medicine, Federal Teaching Hospital, Ido-Ekiti, Nigeria.

2. Olanrewaju Kassim Olasehinde

Department of Community Medicine, Federal Teaching Hospital, Ido-Ekiti, Nigeria.

3. Taofeek Adedayo Sanni

Department of Community Medicine, Federal Teaching Hospital, Ido-Ekiti, and Afe Babalola University, Ado-Ekiti, Nigeria.

4. Ayodeji Andrew Omotoso

Department of Community Medicine, Federal Teaching Hospital, Ido-Ekiti, Nigeria.

5. Rita Omobosola Alabi

Department of Community Medicine, Federal Teaching Hospital, Ido-Ekiti, Nigeria.

6. Paul Oladapo Ajayi

Department of Community Medicine, Ekiti State University, Ado-Ekiti, Nigeria.

7. Kayode Rasaq Adewoye

Department of Community Medicine, Federal Teaching Hospital, Ido-Ekiti, and Afe Babalola University, Ado-Ekiti, Nigeria.

8. John Olujide Ojo

Department of Community Medicine, Federal Teaching Hospital, Ido-Ekiti, and Afe Babalola University, Ado-Ekiti, Nigeria.

9. Olayinka Oloruntoba

Department of Surgery, Federal Teaching Hospital, Ido-Ekiti, Nigeria.

10. Blessing Omobolanle Osho

Department of Public Health, Kwara State University, Malete, Nigeria.

11. Adewumi Rufus Fajugbagbe

Department of Public Health, Kwara State University, Malete, Nigeria.

12. Precious Aderinsola Adeyeye

Department of Public Health, Kwara State University, Malete, Nigeria.

**Formatted:** Left, Space Before: 12 pt, Line spacing: single

**Formatted:** Font: Not Bold

13. Ayotomiwa Fiyinfoluwa Ajayi

Department of Public Health, Kwara State University, Malete, Nigeria.

#### Corresponding Author:

Name: Tope Michael Ipinnimo

Address: Department of Community Medicine, Federal Teaching Hospital, P.M.B 201, Ido-Ekiti, Nigeria.

Phone numbers: +2348062201903

[E-mail address: abbeymagnus@yahoo.com](mailto:abbeymagnus@yahoo.com)

#### Ethical approval and consent to participate:

Ethical approval for the study was sought and obtained from the Ethics and Research Review Committee of the Federal Teaching Hospital, Ido-Ekiti, Nigeria. Informed consent was obtained from all the research subjects.

#### Competing Interest:

Authors declare they have no competing interest in conducting this study

#### Funding:

None

#### Contribution details:

Concept and design- TMI, OKO, TAS Literature search- All Authors

Data collection- All Authors Data analysis- TMI, OKO

Manuscript Preparation- TMI, AAO, ROA, POA Manuscript Editing- All Author

Manuscript Review- All Author

All authors read and approved the final draft of the manuscript.

#### Acknowledgment:

We express gratitude to all the participants of this study.

#### Attitude and Predictors of Exclusive Breastfeeding Practice among Mothers Attending Under-Five Welfare Clinics in a Rural Community in Southwestern Nigeria

**Abstract**

***Background:*** Much~~any~~ previous research on exclusive breastfeeding has focused on urban and semi-urban communities, while there is still a paucity of data from the rural areas. We assessed the attitude and practice of exclusive breastfeeding and its predictors among mothers attending the under-five welfare clinics in a rural community.

***Methods:*** A cross-sectional study was conducted among consecutively recruited 217 mothers attending the three health facilities under-five welfare clinics in Ido-Ekiti, Southwest, Nigeria. Information was collected with a semi-structured interviewer-administered questionnaire which was adapted from previously published research works. Descriptive and inferential statistics were carried out with the aid of IBM SPSS Statistics for Windows, Version 26.0.

***Results:*** More than half of the mothers, 117(53.9%) were ≥30 years, and 191(88.0%) were married. Almost all, 216 (99.5%) attended an ante-natal clinic however, ~~only~~ 174(80.2%) delivered in the health facility. The respondent’s mean ± SD exclusive breastfeeding attitudinal score ~~for the respondents~~ was 29.94 ± 2.14 (maximum obtainable scores was 36) and the proportion of mothers that practiced exclusive breastfeeding was 40.6%. Married mothers were more likely to practice exclusive breastfeeding than their unmarried counterparts (AOR:6.324, 95%CI:1.809–22.114). The common reasons for not practicing exclusive breastfeeding were work schedule 57(26.3%), cultural beliefs and the need to introduce herbal medicine 32(14.7%), and insufficient breast milk 30(13.8%).

***Conclusion:*** This study revealed a good disposition with a suboptimal practice towards exclusive breastfeeding. Also, being married was a positive predictor of exclusive breastfeeding. Therefore, we recommend policies that will improve exclusive breastfeeding among mothers in rural areas, especially those targeting the unmarried in order to achieve the World Health Organization’s target.

***Keywords:*** Attitude, exclusive breastfeeding, practice, Nigeria, rural

#### Key messages

- The global average of exclusive breastfeeding practice between the years 2015-2021 was 48% while the West Africa region had a prevalence of 35.0% which is 15% less than the World Health Organization recommended target of at least 50.0% in all communities by year 2025.
- This prevalence is even lower among the rural population where the least research that could affect policy and practice on exclusive breastfeeding has been carried out.
- This study which was done in a rural community found that the attitude of the mothers was good, however, the practice of exclusive breastfeeding was still suboptimal. In addition, being married was a positive predictor of exclusive breastfeeding among this population.
- Policies that will improve exclusive breastfeeding among mothers in rural areas were recommended. Furthermore, in-depth research into the factors implicated in the low uptake of exclusive breastfeeding among unmarried women is equally advocated.

#### Introduction

Breastmilk is described as the primary source of nutrition for newborns and provides all the nutritional needs for the first few months of life. [1] ~~ideal food for infants because of its contents~~ ~~and safety. [1]~~ It is safe, clean and essentially contains water, nutrients, and antibodies in adequate proportions to promote child growth and development. [~~1,~~2] Exclusive breastfeeding (EBF) involves feeding only breastmilk to a child for the first six months of life, ~~with the~~ ~~exception of~~except for medically prescribed drugs or supplements. [~~1,~~3] ~~Optimal nutrition in the~~ ~~first two years of life, including~~ The World Health Organisation (WHO) recommends EBF ~~and~~ ~~introduction of nutritionally adequate and safe complementary foods by six months of age, is~~ ~~highly recommended~~ because it ~~has been found to~~is cost-effective , and significantly lower the risk of diarrhoea, malnutrition as well as morbidity and mortality among~~in~~ the under-five age group. It has also been found to reduce the risk of diarrhoea and chronic disease in children and ~~to encourage better development overall.~~ [2] Breastfeeding not only proves beneficial to child health but also contributes to maternal~~women’s~~ health as well as providing social and economic benefits. [4]

Globally, ~~T~~the ~~global~~ average ~~of~~ EBF practice between ~~the years~~ 2015-2021 was 48%. [5] and ~~A~~ ~~study done~~ in Italy, only 33.3% practiced EBF. ~~showed that~~ ~~m~~More than half of the Italian women had heard about EBF, with the~~a~~ majority of them believing that EBF is important to both mother and child. [6] ~~More than 90% had breastfed their children but only 33.3% had practiced~~ ~~EBF. [6]~~ However, ~~Another~~ a study in Bangladesh, which focused on mothers in rural areas, showed poor knowledge (34.5%) and practice (27.9%) of EBF. [7] ~~A systematic review of~~ ~~studies carried out i~~I In East Africa, a systematic review revealed~~showed~~ that 42% of mothers preferred to feed their infants with breastmilk alone for the first six months of life while 55.9% of them had practiced EBF for at least six months. [8] The West Africa region had a prevalence of 35.0% for EBF~~exclusive breastfeeding~~ between ~~the years~~ 2015-2021, one of the lowest in the world for the same period. [5]

In Nigeria, only 28.7% of babies were exclusively breastfed in the year 2018. [9] This was even lower in the rural population with a report of 24.3%. [9] A study in a semi-urban area of Sokoto state, Northwest Nigeria, indicated that only 31.0% of women practiced EBF. [10] Another study conducted~~arried out~~ in one of the urban centers in Abuja, the Federal Capital Territory of Nigeria showed that 54.4% had practiced EBF despite a remarkable positive attitude that was determined

by the study (70% agreed that EBF was adequate for their children). [11] A similar study in another urban center in Benin, Edo state, South-south Nigeria, showed an “ever-breastfed rate” of 100.0% but only 40.7% practiced EBF. [12] A survey carried out in two tertiary hospitals in Ogun state, Southwest Nigeria, revealed that more than half (58.8%) of the women practiced EBF. [13]

Optimal breastfeeding practices are important in the prevention and management of malnutrition in children. [2] Stunting, which is a form of malnutrition and an indication of poor nutrition beginning in-utero into early childhood, affected an estimated 144 million (21.3%) under-five children globally in 2019 with the highest burdens in Africa and Asia continents. [14] Over one- third (37.0%) of Nigerian children aged 6 - 59 months are stunted and the prevalence of stunting is almost twice as high among children in rural areas (45.0%) as among those in urban areas (27.0%). [9]

Many previous studies on EBF have centered on urban and semi-urban communities while there is still a paucity of data from the rural areas. [10-13] To the best of the researchers’ knowledge, there has not been a study of this nature assessing the attitude and practice of mothers on EBF in our~~the~~ environment. This research was designed to identify the gap that may exist in the attitude and practice of mothers living in this area toward EBF as it may aid strategy development in improving the nutritional status of infants and children living in the communities.

This study aims to determine the attitude, practice and identify the predictors of practice of EBF among mothers attending the under-five welfare clinics in a rural community in Southwest Nigeria. The findings of this work ~~study~~ would contribute to the literature and can help facilitate further research on the subject. Also, it would aid in defining the gaps in women’s attitude to EBF and its practice, especially in the rural areas. This could then serve as a basis for health planning, policy formulation and implementation to improve EBF practice in rural Nigeria in order to meet the World Health Organization (WHO) recommended target of at least 50.0% EBF rate in all communities by 2025. [15]

#### Methods

##### Study area and design

This was a cross-sectional study carried out between 1st and 30th November 2021 in all the under-five welfare clinics in Ido-Ekiti, Southwest, Nigeria. Ido-Ekiti is a town with an estimated population of 37,000. [16] There are three health facilities with under-five welfare clinic in the community which included~~are~~ the Basic Health Centre (BHC), Comprehensive Health Centre (CHC) and the Federal Teaching Hospital (FTH), Ido-Ekiti. The BHC and CHC are primary health facilities,~~y~~ each with a staff strength of less than thirty healthcare workers mainly registered nurses/midwives and community health extension workers. The BHC and CHC under- five welfare clinics provides services such as immunization and growth monitoring on clinic days which is just once in a week and each of them sees an average of 10 to 20 children per clinic day ~~in their clinics~~. The FTH is a tertiary health facility that serves as a referral centre for other health facilities within the environs. The under-five welfare clinic in FTH is located at the

Department of Community Medicine of the hospital and it offers services every day with the exception of weekends (Saturday to Sunday) to an average of 20 children per day. The staff in the clinic consists of community/public health physicians, public health nurses and community health extension workers.

##### Participants, sample size determination and sampling technique

The study population consisted of mothers attending the under-five welfare clinics in the community. The study included all healthy mothers with healthy baby(ies) attending the clinics. Mothers with adopted or fostered babies were excluded from the study. The minimum sample size for the study was determined using Leslie Fisher’s formula. [17] A 95% confidence interval, and 5% degree of accuracy was assumed. A sample size of 217 was ~~gotten~~ obtained after using the prevalence of EBF from a previous study. [18] All the three health facilities were used for the study. The number of mothers selected from the health facilities was determined using proportionate allocation based on the under-five welfare clinic attendance. Eligible mothers attending the clinic were recruited consecutively into the study until the sample size was achieved.

##### Data Collection Methods

The study instrument used to collect information from the women is a 26-item semi-structured interviewer-administered questionnaire which was adapted from previously published research works. [19, 20] The questionnaire obtained data on socio-demographic and other (such as antenatal clinic attendance, place of delivery) variables, attitude towards EBF, practice of EBF and reasons for not breastfeeding exclusively. The questionnaire was pre-tested on a sample (twenty) of mothers in a CHC in another community which were neither analyzed nor included in the study. Necessary adjustments and corrections were made to the questionnaire after the pre- ~~test. The questionnaire administration was done by the~~ rResearchers administered the questionnaire in a scheduled area of the health facilities through face-to-face interviews with the mothers immediately after their clinics.

##### Data Collection Tools

The study instrument used to collect information from the women is a 26-item semi-structured interviewer-administered questionnaire adapted from previously published research works. [19, 20] The questionnaire obtained data on socio-demographic and other (such as antenatal clinic attendance, place of delivery) variables, attitude towards EBF, practice of EBF and reasons for not breastfeeding exclusively. The questionnaire was pre-tested on a sample (twenty) of mothers in a CHC in another community which were neither analyzed nor included in the study. Necessary adjustments and corrections were made to the questionnaire after the pre-test. The items in the study instrument were tested for internal consistency using the Cronbach coefficient alpha test and a score of 0.9 was obtained. Twelve questions assessed the attitude of mothers on EBF. Mothers were asked to rate their responses on a 3-point Likert scale (1 to 3) measuring the intensity of mother’s attitudes. [19] A positive response was rated 3, a neutral rated 2, and a negative rated 1. The attitudinal score of each respondent was estimated by summing their rating with a maximum obtainable score of 36. Exclusive breastfeeding practice was assessed with a

question inquiring if mothers have given only breast milk to the baby for the first 6 months of life without infant formula or any other product unless it was prescribed by a physician.

##### Variables

The independent variables in this study were sociodemographic variables, maternal characteristics such as antenatal clinic attendance, place of delivery and mode of delivery, as well as attitude towards EBF while the dependent variable included the practice of EBF among the mothers.

##### Data management and statistical analysis

The data collected were entered, cleaned and analysed using computer software IBM SPSS (IBM SPSS Statistics for Windows, Version 26.0. Armonk, NY: IBM Corp). Data were summarized in tables using frequencies and percentages. ~~There were twelve questions that assessed the attitude~~ ~~of mothers on EBF. Mothers were asked to rate their responses on a 3-point Likert scale~~ ~~measuring the intensity of mother's attitudes. [19]~~ The attitudinal scores of the participants were calculated and presented using mean and standard deviation. The mean attitudinal scores across each socio-demographic and maternal~~other~~ variables category ~~were~~was compared using the independent Student-T-test. The association between the mother’s characteristics and the practice of EBF was assessed using the Chi-square test. Binary logistic regression analysis was used to determine the predictors of EBF. The logistic regression model was built with variables with p ≤ 0.2 at the bivariate (Chi-square test) level of analysis. P-values <0.05 were~~as~~ considered significant for the inferential statistics.

##### Ethical Considerations

Ethical approval (ERC/2021/10/18/937A) for this study was obtained from the Human Research and Ethics Review Committee of the Federal Teaching Hospital, Ido-Ekiti, Nigeria. Permission was obtained from the officer-in-charge of the BHC and CHC as well as the consultant-in-charge of the FTH clinics. Additionally, written informed consent to participate was obtained from the mothers ~~prior to~~before the interviews. Confidentiality and anonymity were maintained by not collecting personal data such as names and phone numbers from the respondents

#### Results

~~A total of 217 mothers completed the survey questionnaire.~~ Table 1 shows that more than half of the mothers, 117 (53.9%) were ≥ 30 years and more than two-thirds, 148 (68.2%) held a post- secondary school degree. More than three-quarters, 191 (88.0%) and 179 (82.5%) were married and of Yoruba ethnicity respectively. Ninety-four (43.3%) of them were civil servants. The majority ~~of the mother,~~ 216 (99.5%) of mothers attended the ante-natal clinic, out of which 214 (99~~8~~.1~~6~~%) were informed on EBF. Most, 174 (80.2%) delivered within a health facility and 182 (83.9%) deliveries were via vaginal delivery. ~~More, 91~~Ninety-one (41.9%) ~~of the~~ mothers are currently nursing their first child.

Table 2 shows that the mean (standard deviation) EBF ~~attitude~~attitudinal score for all respondents was 29.94 (2.14) out of 36 maximum possible scores. EBF attitudinal~~e~~ scores significantly differed across age (T = -2.844, p = 0.005), level of education (T = -2.273, p =

0.025), ante-natal clinic attendance during the pregnancy of the baby (T = 4.895, p < 0.001), been informed on EBF during ante-natal clinic (T = 14.378, p < 0.001), mode of delivery (T = - 2.773, p = 0.008) and the birth order of the child (F = 9.533, p = 0.001). The attitudinal score was better among mothers aged ≥ 30 years, those with post-secondary education, mothers with antenatal clinic attendance, those informed on EBF, mothers who had caesarian section delivery, and those with higher birth orders. The ~~A~~attitudinal score did not significantly differ across other variables.

Eighty-eight (40.6%) ~~of the~~ mothers practiced EBF and there was a statistically significant association between marital status and practice of EBF (X = 8.993, p = 0.003). A ~~H~~higher proportion of married mothers practiced EBF than unmarried mothers (married: 44.5%; unmarried: 11.5%). There was no statistically significant association between other respondents’ ~~patient~~ variables and the practice of EBF. Binary logistic regression in tTable 4 revealed that married mothers ~~who are married~~ were about 6 times more likely to practice EBF than those who ~~a~~were unmarried (AOR:6.324, 95%CI: 1.809 – 22.114).

According to Figure 1~~table 5~~, the reasons for the mothers not practicing EBF were work schedule 57 (26.3%), cultural beliefs and the need to introduce herbal medicine 32 (14.7%), insufficient breast milk 30 (13.8%), baby gets hungry and thirsty 28 (12.9%), HIV and unplanned pregnancy 19 (8.8%), early and single motherhood 14 (6.5%) and others reasons 4 (1.8%).

#### Discussion

This study assessed the attitude towards EBF, its practice, and predictors among mothers attending under-five welfare clinics in a rural community. The mean attitude score towards EBF for all mothers in this study was 29.9 out of 36 maximum possible scores. This is 83.2% of the maximum obtainable score which depicts a good disposition towards EBF among the mothers. This finding is higher than what was obtained in rural communities of Lagos, Nigeria. [20] ~~This~~ ~~finding~~ However, it is similar to what was found in studies done among lactating mothers in a rural Ghana community and ~~in~~ Ethiopia. [19, 21~~0~~] This level of attitude is good for the mothers, although there is room for improvement.

This study revealed that EBF attitude iwas positively associated with age, as mothers who were 30 years and over had a better attitude than those aged less than 30 years. This finding is consistent with findings from studies in Ondo state, Southwest, Nigeria [22~~1~~] and in the Island of Abu Dhabi, United Arab Emirates. [23~~2~~] Also, mothers who had attended the antenatal clinic during the pregnancy of the baby had a better attitude than mothers who did not attend the clinic. The antenatal clinic provides nutritional counseling and education including the health benefits of EBF and other infant feeding practices as part of the health promotion measures during visits. Unsurprisingly, this exposure not only improves maternal attitude but subsequently brings about the likelihood of better practice as reported in a study done in Rawalpindi. [24~~3~~]

This study found that mothers with a higher level of education had better dispositions toward EBF. Maternal level of education is a significant determinant of infant feeding practices, and this is supported by a study done in Ondo state, Southwest, Nigeria which found that attitude towards EBF is higher among lactating mothers with higher levels of education. [22~~1~~] Mothers with

higher levels of education can understand and quickly realize the benefits of EBF to their infants and are more motivated to practice it. Programs that promote EBF should be adapted to appeal to mothers who have lower levels of education. The maternal attitude of EBF was better among mothers with 2^nd^ birth than those with their first birth. This is similar to the findings in a study done in Ibadan, Southwest, Nigeria. [25~~4~~] This finding is not surprising as multiparous mothers have previously developed a prior experience and skillset which allows for better EBF attitude in subsequent deliveries.

Less than half (40.6%) of mothers in this study practiced EBF. This is similar to a study done in Edo state, South-south, Nigeria with a prevalence of 40.7%. [12] However, it is higher than the findings in previous studies done in Italy (33.3%), Bangladesh (34.5%), Southern Ethiopia (14.9%), Jigawa (26.8%) and Sokoto (31.0%) states, Northern~~west~~, Nigeria (31.0%) [6, 7, 10, 26, 27] and lower than the prevalence recorded from studies conducted in Debre Berhan District, Ethiopia (68.6%), urban centers of Abuja (54.4%), Ikorodu, Lagos (69.4%) and in Ogun State, Southwest, Nigeria (58.8%). [11, 13, 28, 20] Interestingly, the prevalence of practice of EBF in this study aligns with the WHO targets of at least 50.0% rate in all communities whether rural or urban by 2025. [15] Though, close to the WHO target, several barriers still hinder the practice of EBF. Work schedule, cultural beliefs and the need to introduce herbal medicine, insufficient breast milk, baby gets hungry and thirsty, HIV and unplanned pregnancy, early and single motherhood were mentioned by the mothers as barriers to EBF. Some of these barriers have equally been reported in previous studies. [29~~5~~, 30~~26~~]

It was observed from this study that mothers who are married were about 6 times more likely to practice EBF than those who are unmarried. Expectedly, this result is substantiated by another study in Ethiopia. [31~~27~~] This may be due to the responsibility-sharing and support provided by the husband that could aid the practice of EBF. However, this finding contrast that found in a study done among first-time mothers in Ethiopia which revealed that participants who were not married were almost 3-fold more likely to practice EBF compared with married counterpart. [~~28~~32] This difference could be due to birth-related traditional practices of some~~the~~ Ethiopian ~~people~~ where first-time mothers at 8 months gestational age visit their parent’s home in preparation for births, and afterward, stay for 40 days after birth with their infant and never left alone. Against these background, one can deduce that a good support system from husbands of married women or from parents of unmarried women post-delivery makes the practice of EBF more feasible.

#### Strengths and limitations

**Formatted:** Font: Bold

To the best of our knowledge, this is the first study in Ekiti state that assessed the attitude and practice of exclusive breastfeeding among rural mothers. We also identified predictors of exclusive breastfeeding among this population.

However, ~~T~~the~~is~~ study shares the limitations of the cross-sectional study design. In addition, the~~is~~ research is prone to social desirability and recall bias since questions were asked about the past experiences of the mothers on breastfeeding. Lastly, this is a health facility-based study that was carried out in the under-five welfare clinic and might have left out mothers who do not seek

healthcare or ~~that~~ who patronize other institutions such as patent medicine stores, drug hawkers and herbalists for their healthcare.

#### Conclusion and recommendation

**Formatted:** Font: Bold

~~In conclusion, t~~The study revealed a good disposition towards EBF among mothers with less than half (40.6%) breastfeeding their babies exclusively. The common reasons given for not breastfeeding exclusively included work schedule, cultural beliefs and the need to introduce herbal medicine, insufficient breast milk and the baby gets hungry and thirsty. Also, it was found that ~~the~~ married mothers were more likely to practice EBF than unmarried mothers. We therefore, recommend policies that will improve the EBF of mothers in rural areas toward achieving the WHO target. It is also important to carry out further quantitative and qualitative studies to identify factors associated with low uptake of EBF among unmarried women and develop intervention to improve their practice.

#### Key messages

- The global average of exclusive breastfeeding practice between ~~the years~~ 2015-2021 was 48%, while the West Africa region had a prevalence of 35.0%, which is 15% less than the World Health Organization’s recommended target of at least 50.0% in all communities by the year 2025.
- This prevalence is even lower among the rural population where the least research that could affect policy and practice on exclusive breastfeeding has been carried out.
- This study which was done in a rural community found that the attitude of the mothers was good, however, the practice of exclusive breastfeeding was still suboptimal. In addition, being married was a positive predictor of exclusive breastfeeding among this population.
- Policies that will improve exclusive breastfeeding among mothers in rural areas were recommended. Furthermore, in-depth research into the factors implicated in the low uptake of exclusive breastfeeding among unmarried women is equally advocated.

#### Competing Interest:

Authors declare they have no competing interest in conducting this study

#### Supporting information

S1 File. Minimal data set.

S2 File. Ethics committee approval.

#### References

1. World Health Organization. Breastfeeding. 2024. Available from: [https://www.who.int/health-topics/breastfeeding#tab=tab_](https://www.who.int/health-topics/breastfeeding#tab%3Dtab_1)1 [Last accessed 12 January 2024]

**Formatted:** Font: Bold

1. ~~World Health Organization. Breastfeeding: Questions and Answers. 2015. Available~~ ~~from: https:/~~[~~/www~~](http://www.who.int/news-room/questions-and-answers/item/breastfeeding)~~.~~[~~who.int/news-room/questions-and-answers/item/breastfeeding~~](http://www.who.int/news-room/questions-and-answers/item/breastfeeding) ~~[Last~~ ~~accessed 8 February 2023]~~
2. World Health Organization. Infant and young child feeding. 2021. Available from: <https://www.who.int/news-room/fact-sheets/detail/infant-and-young-child-feeding> [Last accessed 8 February 2023]
3. Centre for Disease Control and Prevention. Nutrition: Infant and Toddler nutrition. 2022. <https://www.cdc.gov/nutrition/infantandtoddlernutrition/definitions.html> [Last accessed 8 February 2023]
4. The Innocenti declaration on the protection, promotion, and support of breastfeeding. 1990. Florence, Italy. World Health Organization/United Nation Children’s Fund.
5. United Nation Children’s Fund. Global Database for Exclusive Breastfeeding. 2022. Available from: [https://data.unicef.org/topic/nutrition/breastfeeding](https://data.unicef.org/topic/nutrition/breastfeeding/)/ [Last accessed 8 February 2023]
6. Cascone D, Tomassoni D, Napolitano F, Di Giuseppe G. Evaluation of Knowledge, Attitudes, and Practices about Exclusive Breastfeeding among Women in Italy. Int J Environ Res Public Health. 2019;16(12):2118. DOI: 10.3390/ijerph16122118.
7. Rana MM, Islam MR, Karim MR, Islam AZ, Haque MA, Shahiduzzaman M, Hossain MG. Knowledge and practices of exclusive breastfeeding among mothers in rural areas of Rajshahi district in Bangladesh: A community clinic-based study. PLoS One. 2020;15(5):e0232027. DOI: 10.1371/journal.pone.0232027.
8. Dukuzumuremyi JPC, Acheampong K, Abesig J, Luo J. Knowledge, attitude, and practice of exclusive breastfeeding among mothers in East Africa: a systematic review. Int Breastfeed J. 2020;15(1):70. DOI: 10.1186/s13006-020-00313-9.
9. National Demographic and Health Survey. 2018. National Population Commission. Federal Republic of Nigeria.
10. Oche MO, Umar AS, Ahmed H. Knowledge and practice of exclusive breastfeeding in Kware, Nigeria. African Health Sciences 2011; 11(3): 518 – 523
11. Okoroiwu GIA, Ubosi NI, Aliyu SM, Eya CP. Knowledge, Attitude and Practice of Exclusive Breastfeeding amongst Mothers of Infants in Gwagwalada Area Council, FCT, Abuja, Nigeria. J. Appl. Sci. Environ. Manage. 2021;25(1):127-132. <https://dx.doi.org/10.4314/jasem.v25i1.18>
12. Akpan UJ, Ibadin MO, Abiodun PO. Breastfeeding practices in early infancy in Benin City, Nigeria. Niger J Paed. 2015;42(2): 126 –131. <http://dx.doi.org/10.4314/njp.v42i2.11>
13. Adebayo A, Oluwaseyi O. Breastfeeding Practices among Mothers in Southwest Nigeria. Ethiop J Health Sci. 2020;30(5):697. <http://dx.doi.org/> 10.4314/ejhs.v30i5.8
14. United Nations Children's Fund, World Health Organization & World Bank. 2020. Levels and trends in child malnutrition: UNICEF/WHO/The World Bank Group joint child malnutrition estimates: key findings of the 2020 edition. World Health Organization. Available from: <https://apps.who.int/iris/handle/10665/331621> [Last accessed 4 March 2023]
15. World Health Organization. Global nutrition targets 2025: Policy brief series. 2014. Available from: <https://www.who.int/publications/i/item/WHO-NMH-NHD-14.2> [Last accessed 4 March 2023]
16. City-facts. Ido-Ekiti. 2015. Available from: <https://www.city-facts.com/ido-ekiti> [Last accessed 4 March 2023]
17. Ipinnimo TM, Abodunrin OL, Durowade KA. Relationship between Cost of Care and Medication Adherence among Hypertensive Patients attending a Tertiary Health Facility in Ekiti State, Nigeria. Journal of community medicine and primary health care. 2022; 34(1):38-57. <https://dx.doi.org/10.4314/jcmphc.v34i1.3>
18. Adewuyi EO, Adefemi K. Breastfeeding in Nigeria: a systematic review. Int J Community Med Public Health. 2016;3(2):385-396. [http://dx.doi.org/10.18203/2394-](http://dx.doi.org/10.18203/2394-6040.ijcmph20160421) [6040.ijcmph20160421](http://dx.doi.org/10.18203/2394-6040.ijcmph20160421)
19. Mogre, V., Dery, M., Gaa, P.K. Knowledge, attitudes and determinants of exclusive breastfeeding practice among Ghanaian rural lactating mothers. Int Breastfeed J. 2016;11:12. <https://doi.org/10.1186/s13006-016-0071-z>

**Formatted:** Font: Bold

~~19.~~20. Balogun MR, Okpalugo OA, Ogunyemi AO, Sekoni AO. Knowledge, Attitude, and Practice of Breastfeeding: A Comparative Study of Mothers in Urban and Rural Communities of Lagos, Southwest Nigeria. Niger Med J. 2017;58(4):123-130. doi: 10.4103/nmj.NMJ_289_16.

~~20.~~21. Tadele N, Habta F, Akmel D, Deges E. Knowledge, attitude and practice towards exclusive breastfeeding among lactating mothers in Mizan Aman town, Southwestern Ethiopia: descriptive cross-sectional study. Int Breastfeed J. 2016;11:3. DOI: 10.1186/s13006-016-0062-0.

~~21.~~22. Adegboro JS. Influence of demographic variables on attitude of nursing mothers towards exclusive breastfeeding in Akoko South West Local Government Area of Ondo State, Nigeria. International Journal of Life Science Research Archive. 2022;3(1):031-8. <https://doi.org/10.53771/ijlsra.2022.3.1.0065>

~~22.~~23. Al Ketbi MI, Al Noman S, Al Ali A, Darwish E, Al Fahim M, Rajah J. Knowledge, attitudes, and practices of breastfeeding among women visiting primary healthcare clinics on the island of Abu Dhabi, United Arab Emirates. Int Breastfeed J. 2018;13:26. DOI: 10.1186/s13006-018-0165-x.

~~23.~~24. Ahmad MO, Sughra U, Kalsoom U, Imran M, Hadi U. Effect of antenatal counseling on exclusive breastfeeding. J Ayub Med Coll Abbottabad. 2012;24(2):116-9.

25. Ayinde AO, Adedokun B, Adeloye AI, Agbonjimi LA, Ayinde TO, Ogunode NJ. Birth order and exclusive breastfeeding practices among mothers with two or more children. Ijcsmr. 2021;1(1):1-10. <https://journalofmedical.org/index.php/ijcsmr>

**Formatted:** Font: Bold

26. Ermancho B., Lette A., Tamirat A., Ermeko T. Determinants of exclusive breastfeeding practice in Southern Ethiopia. J. Food Nutr. Popul. Health. 2021;5(4):50.
27. Sabo A, Abba J, Sunusi Usman U, Musa Saulawa I, Alzoubi MM, Al-Mugheed K, Alsenany SA, Farghaly Abdelaliem SM. Knowledge, attitude, and practice of exclusive breastfeeding among mothers of childbearing age. Front Public Health. 2023;11:1277813. doi: 10.3389/fpubh.2023.1277813.

~~24.~~28. Asfaw MM, Argaw MD, Kefene ZK. Factors associated with exclusive breastfeeding practices in Debre Berhan District, Central Ethiopia: a cross sectional community based study. Int Breastfeed J. 2015 Aug 13;10:23. doi: 10.1186/s13006-015- 0049-2.

~~25.~~29. Otoo GE, Lartey AA, Pérez-Escamilla R. Perceived incentives and barriers to exclusive breastfeeding among periurban Ghanaian women. Journal of Human Lactation. 2009;25(1):34-41. doi: 10.1177/0890334408325072.

~~26.~~30. Apanga PA. A review on facilitators and barriers to exclusive breastfeeding in West Africa. Journal of Biology, Agriculture and Healthcare. 2014;4:9–15.

~~27.~~31. Mamo K, Dengia T, Abubeker A, Girmaye E. Assessment of Exclusive Breastfeeding Practice and Associated Factors among Mothers in West Shoa Zone, Oromia, Ethiopia. Obstet Gynecol Int. 2020;2020:3965873. doi: 10.1155/2020/3965873.

~~28.~~32. Ayalew T. Exclusive breastfeeding practice and associated factors among first- time mothers in Bahir Dar city, North West Ethiopia, removed: A community based cross sectional study. Heliyon. 2020;6(9):e04732. doi:10.1016/j.heliyon.2020.e04732

#### Tables

**Table 1** Socio-demographic and other characteristics of the respondents (N=217)

| **Variable** | **Frequency** | **Percent** |
| --- | --- | --- |
|  | **(N= 217)** | **(%)** |
| **Age**  < 30 years | 100 | 46.1 |
| ≥ 30 years | 117 | 53.9 |
| **Level of education**  Secondary | 69 | 31.8 |
| Post-secondary | 148 | 68.2 |
| **Marital status**  Unmarried | 26 | 12.0 |
| Married | 191 | 88.0 |
| **Religion**  Christianity | 174 | 80.2 |
| Islam | 43 | 19.8 |
| **Tribe**  Yoruba | 179 | 82.5 |
| Hausa | 16 | 7.4 |
| Igbo | 22 | 10.1 |
| **Occupation**  Farmer/trader/artisan | 76 | 35.0 |
| Civil servant | 94 | 43.3 |
| Professional | 20 | 9.2 |
| Student | 27 | 12.5~~4~~ |
| **ANC attendance during the pregnancy of this baby**  Yes | 216 | 99.5 |
| No | 1 | 0.5 |
| **If yes, were you informed on exclusive breastfeeding (n=216)**  Yes | 214 | 99.1~~8.6~~ |
| No | 2 | 0.9 |
| **Place of delivery of this baby**  Outside health facility | 43 | 19.8 |
| Within health facility | 174 | 80.2 |
| **Mode of delivery of this baby**  Vaginal delivery | 182 | 83.9 |
| Caesarean section | 35 | 16.1 |
| **Birth order of the baby**  First | 91 | 41.9 |
| Second | 75 | 34.6 |
| 3 and above | 51 | 23.5 |

**Table 2** ~~Association between mothers’ characteristics and e~~Exclusive breastfeeding attitudinal~~e~~ score associated with respondents’ characteristics (N=217)

**Attitudinale score**

**Variable	Mean ± SD	Test	p-value**

**All respondents**	29.94 ± 2.14	-	-

**Age (in years)**	-2.844^T^	**0.005**

< 30 years	29.50 ± 1.956

≥ 30 years	30.30 ± 2.226

**Level of education**	-2.273^T^	**0.025**

Secondary	29.43 ± 2.29

Post-secondary	30.16 ±2.02

**Marital status**	-0.480^T^	0.634

Unmarried	29.76 ± 1.839

Married	29.95 ± 2.180

**Religion**	1.974^T^	0.053

Christianity	30.09 ± 2.007

Islam	29.27 ± 2.529

**Tribe**	0.317^F^	0.729

Yoruba	29.97 ± 2.075

Hausa	30.00 ± 1.591

Igbo	29.59 ± 2.938

**Occupation**	0.427^F^	0.734

Farmer/trader/artisan	29.72 ± 2.194

C/S	30.09 ± 2.374

Professional	30.00 ± 2.000

Student	29.92 ± 0.196

**ANC attendance during the pregnancy of this baby**	4.895^T^	**<0.001**

Yes	29.98 ± 2.034

No	20.00 ± 0.000

**If yes, were you informed on exclusive breastfeeding**	14.378^T^	**<0.001**

Yes	30.00 ± 2.034

No	28.00 ± 0.000

**Place of delivery**	0.571^T^	0.570

Outside health facility	30.11 ± 2.372

Within health facility	29.98 ± 2.083

**Mode of delivery**	-2.773^T^	**0.008**

**Formatted:** Font: Bold

Vaginal delivery	29.78 ± 2.166

Caesarean section	30.74 ± 1.820

**Birth order**	9.533^F^	**0.001**

First	29.45 ± 2.088

Second	30.77 ± 1.713

Third and above	29.56 ± 2.443

SD: Standard deviation, ^T^: Student T test, ^F^: Analysis of variance

**Table 3** Association between mothers’ characteristics and practice of exclusive breastfeeding (N=217)

**Exclusive breastfeeding**

| **Variable** | **Yes (n=88) (%)** | **No (n=129) (%)** | **Test** | **p-value** |
| --- | --- | --- | --- | --- |
| **Age group (years)**  <30 years | 41 (41.0) | 59 (59.0) | 0.015^X^ | 1.000 |
| >30 years  **Level of education**  Secondary | 47 (40.2)  29 (42.0) | 70 (59.8)  40 (58.0) | 0.091^X^ | 0.769 |
| Post-secondary  **Marital status** | 59 (39.9) | 89 (60.1) | 8.993^Y^ | **0.003** |
| Unmarried | 3 (11.5) | 23 (88.5) |  |  |
| Married  **Religion**  Christianity | 85 (44.5)  72 (41.4) | 106 (55.5)  102 (58.6) | 0.249^X^ | 0.729 |
| Islam  **Tribe**  Yoruba | 16 (37.2)  75 (49.1) | 27 (52.8)  104 (58.9) | 3.506^F^ | 0.173 |
| Hausa | 3 (18.8) | 13 (81.3) |  |  |
| Igbo  **Occupation** | 10 (45.5) | 12 (54.5) | 2.257^X^ | 0.529 |
| Farmer/trader/artisan | 32 (42.9) | 44 (57.1) |  |  |
| C/S | 34 (36.2) | 60 (63.8) |  |  |
| Professional | 8 (40.0) | 12 (60.0) |  |  |
| Student  **ANC attendance during the** | 14 (51.9) | 13 (48.1) | <0.001^Y^ | 1.000 |
| **pregnancy of this baby**  Yes | 88 (40.7) | 128 (59.3) |  |  |
| No  **If yes to question 7, were you informed** | 0 (0.0) | 1 (100.0) | 0.207^Y^ | 0.649 |
| **on exclusive breastfeeding**  Yes | 88 (41.1) | 126 (58.9) |  |  |
| No  **Place of delivery of this baby**  Outside HF | 0 (0.0)  15 (34.9) | 2 (100.0)  28 (65.1) | 0.715^X^ | 0.488 |
| Within HF  **Mode of delivery**  Vaginal delivery | 73 (42.0)  74 (40.7) | 101 (58.0)  108 (59.3) | 0.005^X^ | 1.000 |
| Caesarean section  **Birth order**  First | 14 (40.0)  32 (35.2) | 21 (60.0)  59 (54.8) | 2.859^X^ | 0.245 |

| Second | 36 (48.0) | 39 (52.0) |  | |
| --- | --- | --- | --- | --- |
| Third and above  **Mean attitudinale score** | 20 (39.2)  30.09 ± 1.891 | 31 (60.8)  29.82 ± 2.295 | 0.883^T^ | 0.378 |

^X^: Pearson Chi-Square, ^Y^: Continuity correction, ^F^: Fisher’s Exact Test

**Table 4** Predictors of practice of excusive breastfeeding

#### Predictors

#### 95% CI for AOR

|  | **B** | **P-value** | **AOR** | **Lower** | **Upper** |
| --- | --- | --- | --- | --- | --- |
| **Marital Status (**Married) | 1.844 | **0.004** | 6.324 | 1.809 | 22.114 |
| **Tribe** (Hausa) | 0.357 | 0.459 | 1.429 | 0.556 | 3.674 |
| **Tribe** (Igbo) | 1.429 | 0.072 | 4.173 | 0.879 | 19.803 |

B: Intercept, AOR: Adjusted odd ratio, CI: Confidence interval

**~~Table 5~~** ~~Reasons for not practicing exclusive breastfeeding~~

#### ~~Reasons for not practicing exclusive breastfeeding*~~ ~~Frequency~~

**~~(N=217)~~**

#### ~~Percent~~

**~~(%)~~**

~~The baby gets hungry and the child being thirsty~~	28	~~12.9~~

~~HIV and unintended pregnancies~~	19	~~8.8~~

~~Breast milk was insufficient~~	30	~~13.8~~

~~Work schedule~~	57	~~26.3~~

~~Early and single motherhood~~	14	~~6.5~~

~~Cultural belief and the need to introduce herbal medicine~~	32	~~14.7~~

~~Others~~	~~4~~	~~1.8~~

~~*Multiple response~~

**Reasons for not practicing exclusive breastfeeding***

60	57

50

40

32

30

30

28

19

20

14

10

4

0

Work schedule Cultural belief

and the need to introduce herbal medicine

Breast milk

was insufficient

The baby gets	HIV and

Early and

single motherhood

Others

hungry and the unintended

child being	pregnancies thirsty

*****Multiple response

**Figure 1** Reasons for not practicing exclusive breastfeeding (N=217)

Response to Reviewers

# REPLY TO THE REVIEWERS’ COMMENTS

### **Manuscript title:** Attitude and Predictors of Exclusive Breastfeeding Practice among Mothers Attending Under-Five Welfare Clinics in a Rural Community in Southwestern Nigeria

**Manuscript ID:** PONE-D-23-40179 Dear Academic Editor and Reviewers,

Thank you for taking the time to read and review this work, we are grateful for your insightful comments and contributions. Please find the response to each point raised in the table below.

| **Reviewer**  **Number** | **Original comments of the reviewer** | **Reply by the author(s)** |
| --- | --- | --- |
| REVIEWER  1 | - You can include key messages in your final conclusion section | This has been moved to the conclusion section |
|  | - The definition of breastmilk should be rewrite again. - There is a reference repetition. - This is more to the problem situation and it has to be coherent; revise the background section - You didn't show the actual gap of the study area in this section | The definition of breastmilk has been rewritten Reference repetition has been adjusted  The background section has been revised as suggested and the gap in the study area has been highlighted. |
|  | - The multivariate regression is don’t exist to the extent to identify the predictors - The legend of the table description should clearly show the data - It is good to display the multivariate data of what you have stated right here | The multivariate logistic regression was conducted following bivariate analysis. Variable with p ≤ 0.2 at the bivariate (Chi square) level were included and used to build the logistic model.  The legend of table has been revised as suggested.  A graphical method has been employed to display the data. |
|  | - The discussion is not enough. You have to revise additional and similar topics in Ethiopia and other countries you have stated   In General, at this point I can give these comments. | The discussion section has been revised and additional literature from Ethiopia and Nigeria has been included.  Thank you for your comments and suggestions. |
|  |  |  |
| REVIEWER 2 | The title has been over researched in Nigeria, so what did you  add to the existing knowledge or what is your new finding? If you | To the best of our knowledge, this is the first study in Ekiti state that assessed the attitude and practice of exclusive breastfeeding among rural |

|  | can justify this, what about the knowledge component? it should be included in the title or studied as a factor  The introduction section should clearly describe the public health importance of EBF and mother's attitude, it should be based on your objectives, and must show what interventions undertaken and the existing gaps | mothers. We also identified predictors of exclusive breastfeeding among this population.  Thank you for the suggestion. The Introductory section has been revised as suggested. |
| --- | --- | --- |
|  | Regarding to sample size, your sample size is not adequate, and since the sampling technique is non random, it can not be used for generalization.  How do you measure attitude and practice? I think you used mean, what was your reason to use the mean? I haven't seen a clear operational definition. In addition to this using a five point Likert scale is better than 3 point Liker Scale.  It is better to show the dependent and independent variables clearly.  Regarding to ethical issue, how did you address the confidentiality issue? | Thank you for the comment. We used Leslie Fisher’s formula to estimate the minimum sample size and obtained 217. This was proportionately allocated to the facilities involved based on their client strength. However, respondents were recruited consecutively over the study period and this has been detailed in the method section.  An explanation on how “attitude” and “practice” were measured and analyzed has been added under the new subsection named “Data collection tool” within the methods.  Dependent and independent variables are now clearly stated under “Variables” subsection.  Confidentiality and anonymity were maintained by not collecting personal  data such as names and phone numbers from the respondents. This statement has been added to the “Ethical Consideration subsection” |
|  | Result: the statistical analysis is not performed appropriately and adequately, and lacks clarity. For example you said "The mean ± SD exclusive breastfeeding attitudinal score for the respondents was 29.94 ± 2.14 (maximum obtainable scores was 36) and the proportion of mothers that practiced exclusive breastfeeding was 40.6%." What are these numbers? showing the confidence interval is significant. The attitude score is not is not clear, you put only the mean score in table 2 there should be a table which shows the attitude components.  Marital status is the only variable associated with EBF practice, but what were the other variables in binary logistic regression which entered into multivariable logistic regression? | Twelve questions assessed the attitude of mothers on EBF. Mothers were asked to rate their responses on a 3-point Likert scale (1 to 3) measuring the intensity of mother’s attitudes. A positive response was rated 3, a neutral rated 2, and a negative rated 1. The attitudinal score of each respondent was estimated by summing their rating with a maximum obtainable score of 36. The attitudinal scores of all the participants and across different variable categories were calculated and presented using mean and standard deviation.  Exclusive breastfeeding practice was assessed with a question inquiring if mothers have given only breast milk to the baby for the first 6 months of life without infant formula or any other product unless it was prescribed by a physician. This was a dichotomous variable (“Exclusively breastfed” OR “Not exclusively breastfed”).  The logistic regression model was built with variables with p ≤ 0.2 at the bivariate (Chi-square test) level of analysis and these variables included  marital status as well as tribe. This is presented in Table 4 |

|  | Discussion and conclusion should be objective based, and the recommendation should be based on your finding. | Thank you for the comment. This has been revised as suggested. |
| --- | --- | --- |
|  | Additionally, I am not sure that the referencing style fulfils PLOS ONE's criteria. | We have double-checked this to fulfil the PLOS ONE’s criteria |
|  |  |  |
| REVIEWER 3 | The study topic is scientifically sound and may contribute to body knowledge in maternal and child health. However, there are many gross editorial and methodological issues and problems. I have tracked and changed all the comments and suggestions with an attached document.  1. The study was written in standard academic English. | Thank you for the comments. All the corrections under track changes have been made. In addition, we have revised other editorial and grammatical corrections. |
|  | 2. The methodology needs a massive correction. You could not provide a specific Analysis that fits the nature of your data.    a. You must have separate titles for data collection methods, tools, or study measurements. They are district features of the study methodology.    b. If you did a pre-test, what was the tool's reliability?    c. you need to state explicitly how you measured the exclusive breastfeeding outcome variable.    d. What is the need to use chi-square? Do you have a specific justification? You also used binary logistic regression, which measures the association between dependent and independent variables more accurately than chi-square. Table 2 also indicates you used Yate’s continuity correction and Fisher’s exact test.    e. Did you conclude the association between dependent and independent variables only based on the result of binary logic regression? This is not scientifically sound.   Table 1 depicts. You used Analysis of variance and T-test. So, you did not select one appropriate analysis method for this study. If you have justification, why do you use a mix of different | This methods section has been revised based on your suggestions. Data collection method is now separate from the Study instrument.  Tool reliability has been included.  An explanation on how “attitude” and “practice” were measured and analyzed has been added under the new subsection named “Data collection tool” within the methods.  The Chi-square test was used to compare the association between the practice of EBF and sociodemographic/maternal variables at the bivariate level of analysis. It was used because they were both categorical variables and Chi-square is used to test for association between two categorical variables. The Yate correction and Fisher’s exact test were used in situations where we have observation in cell(s) < 5 as shown that the Chi- square test may not be the best test in such situations.  The binary logistic regression analysis was used at the multivariate level of analysis to determine the predictors of EBF. The logistic regression model was built with variables with p ≤ 0.2 at the bivariate (Chi-square test) level of analysis.  Student-T-test and ANOVA (F-test) were used to compare the attitudinal scores as these were continuous variables (mean and SD). T test was for comparing 2 groups while ANOVA was for >2 groups. |

|  | analyses to justify it? |  |
| --- | --- | --- |
|  | 3. You need to add the strengths and limitations of the study and the conclusion and recommendation sections. | Done as suggested |
