# Supplementary material for: Attitude and predictors of exclusive breastfeeding practice among mothers attending under-five welfare clinics in a rural community in Southwestern Nigeria
Source: PLoS One. 2024 Mar 28;19(3):e0299843. doi: 10.1371/journal.pone.0299843 (PMC10977687; doi:10.1371/journal.pone.0299843)
Supplement: Attachment — Submitted filename: R2.Response to Reviewers - Breastfeeding in Ido-Ekiti.doc [file pone.0299843.s006.doc]

**REPLY TO THE REVIEWERS’ COMMENTS**

**Manuscript title:** Attitude and Predictors of Exclusive Breastfeeding Practice among Mothers Attending Under-Five Welfare Clinics in a Rural Community in Southwestern Nigeria

**Manuscript ID:** PONE-D-23-40179

Dear Academic Editor and Reviewers,

Thank you once again for your comments and suggestions. Please find the response to each point raised in the table below.

| **Reviewer Number** | **Original comments of the reviewer** | **Reply by the author(s)** |
| --- | --- | --- |
| REVIEWER 1 | More or less, the authors addressed the comments, and the author amended them accordingly. There are some modifications that the author didn’t consider changing. The introductory sentence should be included in the background data. There is still a gap in justifying the actual study area. | Thank you for your comment.  The study area was justified using the statement in paragraph 5 of the introduction: “Many previous studies on EBF have centered on urban and semi-urban communities, while there is still a paucity of data from rural areas. [10-13] To the best of the authors’ knowledge, there has not been a study of this nature assessing the attitude and practice of mothers on EBF in our environment. This research was designed to identify the gap in the attitude and practice of mothers living in this area toward EBF as it may aid strategy development in improving the nutritional status of infants and children living in the communities.” |
|  | Results and discussion It is better to report the mean age of mothers  One of the variables in the result section is work schedule, and the majority of the respondents are farmers. How do you justify this? Similar comments were made during the discussion as well. | The mean age of mothers has been included in Table 1 and the prose of the result section.  From the study, the majority of the respondents were not farmers. Close to half of the respondents were civil servants (94, 43.3%), and only about one-third were farmers/traders/artisans (76, 35.0%). The “work schedule” was the most (57, 26.3%) prevalent reason for not breastfeeding exclusively by the mothers, and only mothers who didn’t practice exclusive breastfeeding gave reasons. Additionally, it could be seen from the subgroup analysis in Table 3, that the civil servant was the subgroup with the highest proportion (as well as absolute number) of mothers who did not practice exclusive breastfeeding. The majority of the reasons were most likely from them. |
| REVIEWER 3 | General comments and suggestions. The comments and suggestions are not adequately addressed. There are areas that have needed major revision. For instance, the Methodology, result, discussion, and recommendations sections. Additionally, there are editorial issues. | Thank you for the comments. All editorial issues highlighted under track changes have been revised as suggested.  Issues on methods, results, discussion, and recommendations have been addressed and the response to each of the points raised is found below |
|  | Methodology: Variable page 8. You treat Attitude as the dependent variable in the result section. See Table 2. It is so confusing. | Attitude is a dependent variable and this has been corrected under the “Variables subsection” of the methods |
|  | Methodology: Data management and statistical analysis. Yes, you calculated the mean and standard deviation based on the attitudinal score for each independent variable. The question is: Is attitude toward breastfeeding not a dependent variable? The second question is, do you understand when we can use T-test and ANOVA? Because to use a T-test, you need to have at least two Mean scores. For Anova, it is supposed to be three means or more. You have a single population. You collected data at a single point. You did not have a standard attitudinal score to compare with. So, your statistical analysis selection reflects a poor understanding of this method. I Attached a document for your understanding. | T-test and ANOVA (F-test) were used to compare the attitudinal scores as these were continuous variables (mean and standard deviation).  As mentioned, the T-test is used to compare 2 group means while ANOVA is used for >2 group means. We indeed have a single population; however, these tests were utilized in subgroup analysis where the participants were grouped using their sociodemographic as well as other characteristics. Depending on the number of categories within the characteristics, we then used either   1. Student’s t-test (if only 2 categories) e.g Age group (<30 yrs/≥30yrs)   OR   2. ANOVA (If > 2 categories) e.g Tribe (Yoruba/Hausa/Igbo).   **For example**: In Table 2, using age, we grouped mothers into those below 30 years and those 30 years and above. We estimated the mean (±SD) attitudinal scores of mothers for each of the two groups and compared them using an independent student’s t-test.  **Another example**: Also in Table 2, using tribe, we grouped mothers into Yoruba, Hausa, and Igbo. We estimated the mean (±SD) attitudinal score of mothers in each of the 3 tribe groups. In this case, we compare the means using ANOVA.  There was indeed no standard population to compare the overall score with, hence we did not use a one-sample t-test but instead, an independent student t-test was employed for the subgroup analysis.  Please see an article explaining the statistical concept employed in this analysis:  Mishra P, Singh U, Pandey CM, Mishra P, Pandey G. Application of student's t-test, analysis of variance, and covariance. Ann Card Anaesth. 2019 Oct-Dec;22(4):407-411. doi: 10.4103/aca.ACA_94_19. PMID: 31621677; PMCID: PMC6813708. |
|  | Result Page 10. The logical order of the paragraphs is not correct. Some information is fabricated. | Thank you for the comment. This logical order has been revised as suggested.  No information is fabricated in this study as all findings reported are from the collected data. The table that contains the information has been cited in the prose. Also, I have included a new row “All respondents” in Table 3 for emphasis and to show clarity. |
|  | Discussion: page 10 paragraph 6. EBF attitude was not stated as the dependent variable in the methodology section. So, it is difficult to discuss here. Additionally, you used a T-test to identify associated factors to EBF attitude, which is an inappropriate and unacceptable choice of analysis. Review this thoroughly. (for reference table 2.) | EBF attitude has been included as a dependent variable.  The choice and reason behind the statistical analysis have been explained above with reference. |
|  | Discussion: page 11 paragraph 2. Where did you find this finding? Education is not in the final model. Education is not significantly associated with the practice of exclusive breastfeeding in the Chi-square test (Reference Table 3) | This finding is within Table 2 i.e “EBF attitudinal score associated with respondents’ characteristics”. Education is significantly associated with the attitude toward EBF (T = -2.273, p = 0.025). |
|  | Discussion: page 11 paragraph 3. First, discuss descriptive findings before analytic ones. Follow the logical order. | This was done as suggested. However, attitude toward EBF was first discussed in full before the Practice of EBF. |
|  | Discussion: page 11 paragraph . A logistic regression model with a single variable cannot predict the practice of exclusive breastfeeding. This is not utterly acceptable. (reference table 4) | Thank you for the comment once again. The binary logistic regression analysis was not with a single variable. The logistic regression model was built with variables with p ≤ 0.2 at the bivariate (Chi-square test) level of analysis. Two variables fulfilled this criterion which were **Marital status** and **Tribe**. Both were included in the model and are enough (minimum of 2 independent variables) for logistic regression. For clarity and better understanding, I have added the reference categories to each of the variables in Table 4. |
|  | Conclusion and recommendation: page 12 paragraph1. These are descriptive findings from this study. However, factors associated with exclusive breastfeeding are more reliable and credible for recommending solutions. It is not conclusive to recommend unless these factors are statistically significant with exclusive breastfeeding practice. (reference Figure 1) Hence, this study's analytical recommendation should come from analytical findings. | I have removed the descriptive findings and left the statistically significant EBF factor as suggested. |
